# Vitamin D, immune microenvironment, and cervical lesions: mechanisms and therapeutic strategies from polyps to carcinoma

**DOI:** 10.3389/fnut.2025.1688910

**Published:** 2025-10-31

**Authors:** Zheng He, Cheng Du

**Affiliations:** Department of Obstetrics and Gynecology, Shengjing Hospital of China Medical University, Heping District, Shenyang, Liaoning, China

**Keywords:** VITD, immune microenvironment, cervical lesions, human papillomavirus, therapeutic targets

## Abstract

Persistent infection with high-risk human papillomavirus (HPV) together with progressive dysregulation of the cervical tumor immune microenvironment (TIME) drives the continuum from cervical intraepithelial neoplasia (CIN) to invasive cancer. Vitamin D (VitD) signaling via the vitamin D receptor (VDR) intersects this trajectory by inducing antimicrobial peptides, strengthening epithelial barrier function, redirecting dendritic cells (DCs) toward less inflammatory programs, attenuating Th1 and Th17 activity, and promoting regulatory T-cell responses. These coordinated effects can shift a “cold” cervical niche toward improved viral clearance and controlled inflammation. Clinically, a randomized trial reported that biweekly cholecalciferol at 50,000 IU for 6 months increased CIN1 regression to 84.6%. Preclinical and early clinical studies also suggest that VitD enhances radiotherapy (RT) responses by suppressing autophagy, promoting apoptosis, and reducing the neutrophil-to-lymphocyte ratio (NLR). Translational options include systemic supplementation with monitoring of 25-hydroxyvitamin D (25 [OH]D), cervicovaginal delivery to concentrate drug at lesion sites, and development of low-calcemic VDR agonists used alongside standard antiviral and oncologic care. Key uncertainties remain, including tissue heterogeneity of VDR expression, optimal dosing windows and target 25(OH)D ranges for cervical endpoints, and safety at higher exposures such as hypercalcemia. This review aims to integrate mechanistic and clinical evidence, define stage-specific roles of the VitD–VDR axis across the CIN–cancer spectrum, and outline practical strategies and research priorities for VitD-based adjunctive interventions in HPV-associated cervical disease.

## 1 Introduction

Cervical cancer ranks as the fourth most common malignancy and the fourth leading cause of cancer-related mortality among women worldwide (1, 2). Its primary etiological mechanism involves persistent infection with high-risk human papillomavirus (HPV), which induces cervical intraepithelial neoplasia (CIN) and subsequently progresses to invasive cervical carcinoma (3, 4). Although most HPV infections are transient and self-limiting, a subset of individuals fails to clear the virus due to immune surveillance defects or viral immune evasion, resulting in the progression of CIN from low-grade lesions (CIN1 or LSIL) to high-grade lesions (CIN2/3), and eventually to invasive cancer (5, 6).

Recent evidence highlights the central role of host immunity in cervical carcinogenesis beyond its traditional antiviral function. The cervical tumor immune microenvironment (TIME) remodels across disease progression, shifting from an immune-active state in early lesions to a strongly immunosuppressive milieu in advanced disease (7). Hallmarks include impaired antigen presentation, exhaustion of effector T cells, and enrichment of regulatory T cells and M2-polarized macrophages (8). Acting together with persistent high-risk HPV, these immune changes serve as a secondary oncogenic driver that accelerates malignant transformation. Accordingly, strategies that reprogram the local immune microenvironment are gaining prominence in prevention and treatment of cervical cancer (9, 10).

Vitamin D (VitD) (particularly its active form, 1,25-dihydroxyVitD_3_ (VD_3_) is traditionally known for its essential role in calcium and bone metabolism. However, it is now widely recognized as a critical immunomodulatory molecule (11). Through interaction with the vitamin D receptor (VDR), VD_3_ influences the functional phenotype of diverse immune cell subsets, enhancing innate immune defenses, suppressing pro-inflammatory responses, and promoting immune tolerance (12). Epidemiological studies have revealed that individuals with VitD deficiency exhibit a higher susceptibility to high-risk HPV infection and demonstrate reduced rates of CIN regression (13). At the tissue level, downregulation of VDR expression in cervical cancer specimens is closely associated with elevated expression of immune exhaustion markers, implicating the VD–VDR signaling axis as a potential regulator of TIME remodeling (14, 15).

This review systematically examines how VitD shapes the cervical TIME across the disease continuum, from cervical polyps and CIN to invasive carcinoma. We focus on mechanisms that influence innate barrier integrity, dendritic cells (DCs) maturation, T-cell polarization, and immune-escape pathways, and we assess the translational potential of targeting the VitD–VDR axis as a preventive or adjunctive therapeutic strategy. By linking these mechanistic insights to stage-specific biology, we provide a conceptual framework and therapeutic rationale for precision interventions in HPV-associated cervical carcinogenesis.

## 2 Cervical lesion progression and immune microenvironmental features

Cervical lesions typically follow a continuum from cervical polyps to CIN1/2/3, and eventually progress to invasive cervical carcinoma. Each stage of lesion development is characterized by distinct alterations in the local TIME, which critically influence disease trajectory.

### 2.1 Cervical polyps

Cervical polyps are commonly regarded as benign hyperplastic lesions resulting from prolonged chronic inflammation and are frequently observed in multiparous women (16). While polyps are largely non-malignant, their association with persistent inflammatory stimuli may increase the risk of subsequent cervical pathologies. The development of cervical polyps is linked to prolonged exposure of the cervical mucosa to bacterial infections, viral insults (including low-risk HPV), or mechanical irritation (17).

Histologically, polyps are characterized by fibrous stromal hyperplasia with prominent capillary dilation, and the epithelial surface may exhibit squamous or columnar epithelial proliferation. The cervical tissue frequently exhibits chronic inflammatory infiltration, predominantly by lymphocytes, plasma cells, and neutrophils (18). Occasional detection of DCs and macrophages suggests active immune surveillance and local immune engagement.

The chronic inflammatory microenvironment of polyps is marked by elevated pro-inflammatory cytokines. These factors promote angiogenesis and stromal proliferation while potentially impairing mucosal barrier function, thereby increasing susceptibility to secondary infections like HPV (19). Notably, innate immune components such as macrophages and DCs are often hyperactivated, whereas adaptive immune responses, particularly those mediated by T cells for viral clearance, are suppressed or functionally impaired. This immune imbalance may create a permissive microenvironment that facilitates progression toward higher-grade cervical lesions.

### 2.2 Low-grade squamous intraepithelial lesions (CIN1)

CIN1 represents mild cervical epithelial dysplasia, most commonly induced by transient infection with high-risk HPV subtypes (e.g., HPV16, HPV18) or occasionally low-risk types. Approximately 60–80% of CIN1 cases undergo spontaneous regression owing to host immune surveillance (20, 21). At this stage, the TIME is generally “immune-active,” characterized by infiltration of effector T cells and DCs.

CIN1 lesions are typically infiltrated by abundant CD8^+^ cytotoxic T lymphocytes, CD4^+^ helper T cells and Langerhans-type DCs, reflecting an active immune response directed against HPV-infected epithelial cells (22). A Th1-polarized immune profile predominates, with upregulation of pro-inflammatory cytokines such as IFN-γ and IL-2. This immune activation facilitates viral clearance, apoptosis of infected cells, and epithelial repair (23).

However, a subset of CIN1 patients displays a slightly TIME, evidenced by the infiltration of Treg cells, increased M2 macrophages, and a cytokine milieu skewed toward IL-10 and TGF-β. This mild immunosuppressive state may impair timely viral clearance, increasing the risk of progression to higher-grade lesions (CIN2/3) (24).

Thus, the TIME in CIN1 represents a dynamic equilibrium in which effective immune activation promotes lesion regression, whereas mild immune suppression may predispose to disease progression.

### 2.3 High-grade squamous intraepithelial lesions (CIN2/3)

CIN2 and CIN3 are considered moderate to severe dysplastic lesions and represent critical precancerous stages, often driven by persistent high-risk HPV infection. The immune microenvironment in these lesions transitions from an “immune-active” to an “immune-silent” or even “immune-excluded” state (25).

In CIN2/3 lesions, effector T cells, including CD8^+^ CTL and Th1 helper cells, are significantly diminished, whereas FoxP3^+^ Tregs are markedly elevated, resulting in a predominantly TIME. Macrophage polarization shifts toward a tumor-promoting M2 phenotype, which secretes immunosuppressive mediators such as IL-10, VEGF, and Arginase-1 (26). These factors inhibit effector T cell activity and enhance immune escape and neovascularization.

Additionally, dendritic cell function becomes compromised, with phenotypic changes toward a tolerogenic profile and diminished antigen-presenting capacity. NK cell infiltration and cytotoxic function are also suppressed, further weakening local immune surveillance (27). Cytokine profiles demonstrate reduced levels of pro-inflammatory mediators and increased levels of inhibitory cytokines (28).

Furthermore, expression of immune checkpoint molecules is progressively upregulated in CIN2/3, contributing to T cell exhaustion and diminished antitumor immunity (29). These alterations collectively signify the emergence of immune escape mechanisms at the precancerous stage, which, if not effectively controlled, may facilitate the progression to invasive cervical cancer.

Single-cell RNA sequencing (scRNA-seq) and spatial transcriptomics (ST) delineate a stage-specific reprogramming of the cervical tumor immune microenvironment at CIN2/3 that anticipates invasion. Cytotoxic CD8^+^ T cells contract and acquire exhaustion signatures marked by PD1, TIGIT and TOX, with reduced granzyme programs and a narrowing of T-cell receptor clonotypes. Regulatory T cells expand and express CCR4 and CTLA4, while the CXCL9 and CXCL10 chemokine axis that supports CXCR3^+^ effector trafficking declines. Myeloid compartments shift toward immunoregulatory states typified by SPP1^+^ macrophages with high IL10, VEGFA and ARG1, accompanied by a loss of CCR7-programmed migratory dendritic cells and attenuation of cross-presentation modules associated with BATF3-dependent conventional dendritic cells (30). Epithelial trajectories inferred by pseudotime place proliferative basal cells on a branch characterized by partial epithelial–mesenchymal transition, activation of TGF-β and hypoxia programs, and down-modulation of antigen-processing and MHC class I pathways including B2M and TAP1. Spatial maps localize PD-L1 expression to epithelial nests and adjoining stroma and reveal immune exclusion patterns in which Tregs and SPP1^+^ macrophages form a perilesional ring separated from epithelial clusters by collagen-rich, ACTA2 and COL1A1-high fibroblasts (31).

Ligand–receptor analyses identify recruitment and suppression circuits that consolidate immune escape at this stage. CCL22 and CCL17 produced by myeloid and stromal cells engage CCR4 on Tregs. PD-1 with PD-L1 and CTLA4 with CD80 or CD86 enforce T-cell dysfunction. SPP1 with CD44 and MIF with CD74 support protumor myeloid signaling, while TGFB1 with TGFBR1 or TGFBR2 sustains fibroblast activation and matrix remodeling. Endothelial cells display tip-like states with VEGFA and KDR signaling and organize perivascular immune aggregates that lack effective cytotoxic programs (32). Collectively, these single-cell and spatial data portray CIN2/3 as a tipping point at which exhausted T-cell states, immunosuppressive myeloid programs and fibrovascular remodeling converge to create an immune-excluded niche that lowers the barrier to invasion (33).

Targeting the immunosuppressive TIME at the CIN2/3 stage may offer a critical opportunity to prevent cervical cancer development.

### 2.4 Invasive cervical cancer

As lesions progress to invasive cervical cancer, the tumor further consolidates an immune-evasive microenvironment. Cervical tumors frequently express high levels of immunosuppressive molecules such as PD-L1 and IDO-1, leading to T cell exhaustion and immune tolerance. Treg infiltration is markedly increased, accompanied by elevated production of TGF-β, which inhibit effector T cell infiltration and induce functional impairment (34).

TAMs predominantly display an M2-polarized phenotype, releasing immunosuppressive mediators that promote tumor growth and immune escape (35). Overall, the TIME in invasive cervical cancer is characterized by “immune cold” or “immune excluded” features, in stark contrast to the “immune-active” profile observed in early CIN1 lesions (36).

This progressive immunologic transformation illustrates the immune escape trajectory across cervical lesion development: persistent viral antigen exposure and tumor selection pressures drive the transition from immune activation to immune suppression and exhaustion, ultimately permitting tumor cells to evade immune surveillance (37).

High-throughput genomics and transcriptomics refine the immune portrait of invasive cervical cancer and clarify why immune evasion becomes consolidated at this stage. Whole-exome and whole-genome studies show a mutation spectrum dominated by APOBEC signatures with recurrent alterations in PIK3CA, FBXW7, EP300, and STK11, together with copy-number gains on 3q and 5p and focal losses in antigen presentation regions (38). Viral–host integration events create enhancer hijacking and viral–host chimeric transcripts that reprogram interferon signaling and cell cycle control and are associated with immune checkpoint upregulation. Bulk RNA-seq identifies immune classes ranging from interferon-inflamed to mesenchymal and immune-excluded phenotypes. The latter display downregulation of antigen processing genes such as B2M and TAP1, enrichment of TGF-β and hypoxia programs, and matrix remodeling that spatially restricts effector trafficking (39). TCR sequencing reveals contraction of repertoire diversity with expansion of exhaustion-biased clonotypes, consistent with progressive loss of cytotoxic function. scRNA-seq and ST demonstrate accumulation of exhausted CD8 T cells marked by LAG3 and TOX, expansion of FOXP3 positive regulatory T cells, dominance of SPP1 positive macrophages with IL10, VEGFA and ARG1 programs, attenuation of CCR7 dependent migratory DCs, and activation of collagen producing cancer-associated fibroblasts that form barriers to immune entry (40). Spatial ligand–receptor inference highlights CCL22 or CCL17 to CCR4 and SPP1 to CD44, interactions that together reinforce immune suppression and exclusion. DNA methylation and chromatin accessibility profiling add an epigenetic layer by showing promoter hypermethylation and closed chromatin at antigen presentation and chemokine loci, with parallel activation of remodeling factors in stroma (41). Integrating these data suggests composite biomarkers that outperform single markers for immunotherapy selection, including combinations of APOBEC activity or tumor mutational burden, HLA class I loss, exhausted T-cell state scores, SPP1 positive macrophage fraction, stromal TGF-β signaling and epithelial PD-L1 expression (42).

Understanding and intervening in this immunologic evolution is essential for developing effective strategies for cervical cancer prevention and treatment.

## 3 Overview of VitD/VDR signaling and immunoregulatory mechanisms

VitD is a group of fat-soluble secosteroids primarily composed of cholecalciferol (VitD_3_) and ergocalciferol (VitD_2_), derived from diet or synthesized in the skin. Among them, VitD_3_, produced by ultraviolet B-induced conversion of 7-dehydrocholesterol in the skin, constitutes the major source in humans (43). VitD itself has limited biological activity and requires two sequential hydroxylation steps for activation. First, in the liver, it is converted to 25-hydroxyVitD [25(OH)D] by 25-hydroxylases such as CYP2R1 and CYP27A1 (44). Subsequently, in the renal proximal tubules and various peripheral tissues, 25(OH)D is converted by 1α-hydroxylase (CYP27B1) to the biologically active form, 1,25-dihydroxyVitD [1,25(OH)_2_D, also known as calcitriol] (45).

**Figure 1:**
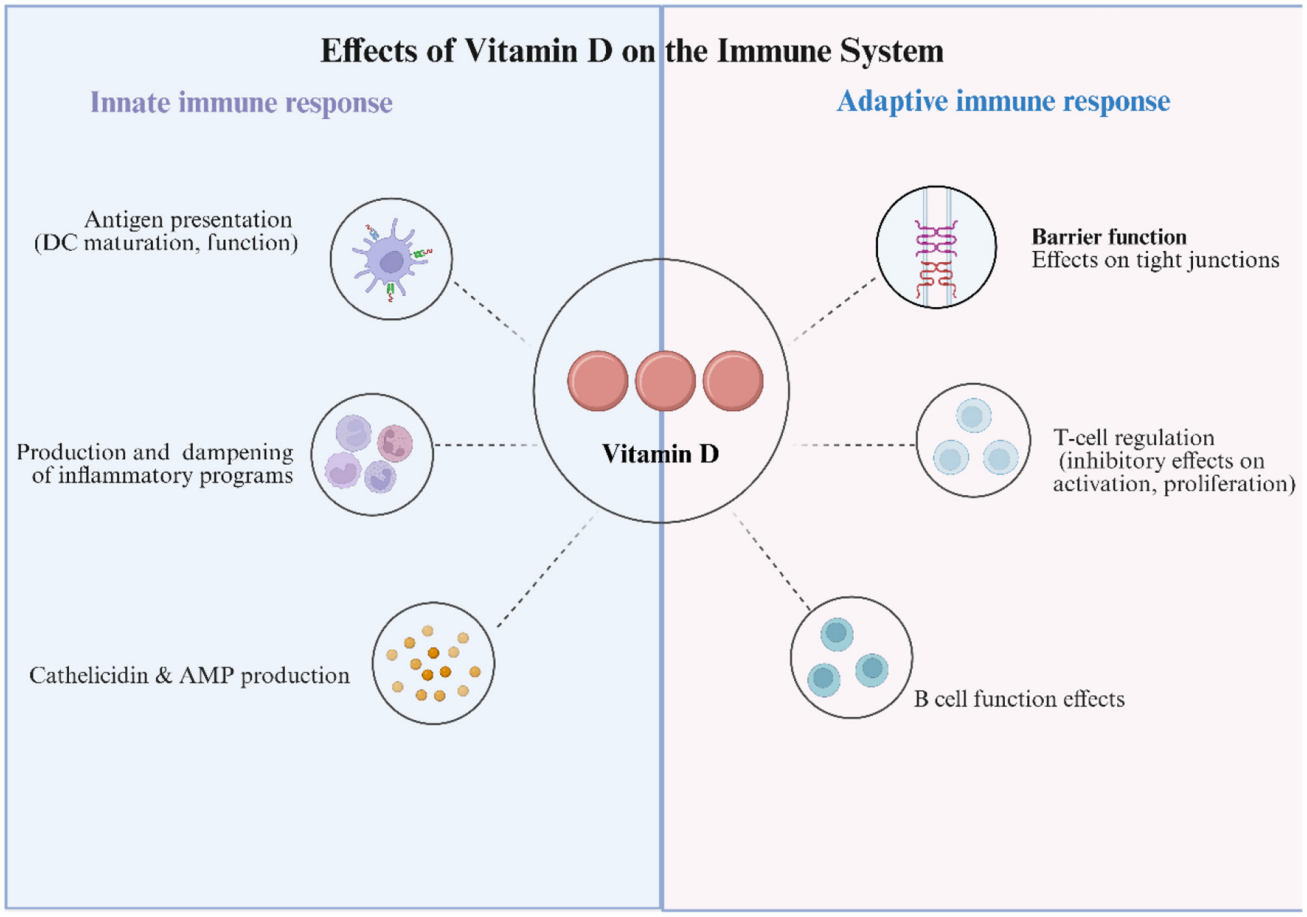
Effects of Vitamin D on the Immune System. Schematic summarizing the immunomodulatory actions of 25(OH)D and its active metabolite 1,25(OH)_2_D via vitamin D receptor (VDR) signaling. Innate immune: Vitamin D (VitD) calibrates antigen presentation by promoting a more tolerogenic dendritic-cell (DC) phenotype; dampens pro-inflammatory programs in monocytes/macrophages/neutrophils (e.g., down-modulates NF-κB and inflammasome activity; ↑IL-10, ↓IL-12/IL-6/TNF); and induces antimicrobial peptides (AMP), notably cathelicidin and β-defensins. Adaptive immune: VitD strengthens epithelial barrier function by upregulating tight-junction proteins (claudins/occludin); modulates T-cell responses by attenuating activation and proliferation, suppressing Th1/Th17 polarization, and favoring Treg differentiation; and shapes B-cell functions, influencing proliferation, differentiation, and antibody production. Collectively, VitD tempers excessive inflammation while supporting mucosal defense—mechanisms relevant to HPV control and cervical mucosal homeostasis.

Calcitriol exerts its effects by binding to the VDR, a member of the nuclear receptor superfamily, which is broadly expressed across multiple tissue types, including immune cells such as T cells, B cells, DCs, and macrophages (Figure 1). Upon ligand binding, VDR heterodimerizes with RXR and binds to VDREs in the promoter regions of target genes, recruiting transcriptional machinery to regulate gene expression (46). Through this genomic pathway, VitD modulates a wide range of genes involved in cellular proliferation, differentiation, and immune regulation. In addition, membrane-associated VDR can mediate rapid, non-genomic signaling. However, the immunomodulatory effects of VitD are primarily mediated via transcriptional regulation of cytokines and immune effector molecules.

### 3.1 Regulation of innate immunity by VitD

Innate immune cells, upon recognizing pathogen-associated molecular patterns (PAMPs), rapidly upregulate both CYP27B1 and VDR via Toll-like receptors (TLRs), including TLR2, TLR4, and TLR9 (47). This enables the local conversion of circulating 25(OH)D to 1,25(OH)_2_D at sites of infection or inflammation, initiating transcriptional and non-transcriptional responses that enhance antimicrobial defenses, stabilize epithelial barriers, and limit inflammatory damage—without significantly perturbing systemic calcium homeostasis (48).

#### 3.1.1 Macrophages and monocytes

Activated macrophages and monocytes, under the control of the CYP27B1-VDR axis, upregulate the expression of CAMP, DEFB2, inducible nitric oxide synthase (iNOS), and the iron-regulatory peptide hepcidin. The resulting antimicrobial peptides, including cathelicidin LL-37 and β-defensin-2, exert broad-spectrum activity by disrupting bacterial and fungal membranes and contribute to antiviral defense by targeting viral envelopes (49). Nitric oxide and hepcidin further restrict pathogen proliferation via oxidative stress and iron sequestration. Additionally, 1,25(OH)_2_D can act in an autocrine or paracrine fashion to modulate neighboring T and NK cell responses (50). However, when circulating 25(OH)D levels exceed 75 nmol/L, excessive extrarenal synthesis of 1,25(OH)_2_D by activated macrophages may disrupt calcium homeostasis, potentially resulting in hypercalcemia (51). This phenomenon has been observed in patients with granulomatous diseases, particularly during the summer months. To prevent immune–metabolic imbalance, macrophages induce the inactive splice variant CYP24A1-SV and downregulate TLRs, forming a localized negative feedback loop.

#### 3.1.2 Antigen-presenting cells and natural killer (NK) cells

In DCs, 1,25(OH)_2_D suppresses CIITA and NF-κB signaling, maintaining cells in an immature, tolerogenic state characterized by reduced MHC class II and CD80/86 expression, decreased IL-12, and increased IL-10 production. This attenuates Th1/Th17 polarization (52). Similarly, monocytes exhibit downregulation of TLR2/4/9 and pro-inflammatory cytokines, effectively raising the threshold for autoimmunity (53). The effects of 1,25(OH)_2_D on NK cells appear to be context-dependent. Under conditions of high inflammatory burden, NK cell cytotoxicity and IFN-γ production are suppressed, whereas in low-inflammatory settings, mild activation may be observed (54). These findings suggest a bidirectional regulatory role for 1,25(OH)_2_D that is relevant in both infectious and malignant contexts.

Neutrophil extracellular traps (NETs) are web-like chromatin structures extruded by activated neutrophils and decorated with histones, myeloperoxidase, neutrophil elastase, and antimicrobial peptides such as LL-37. NET formation is induced by pathogen sensing and sterile danger signals through pathways that involve NADPH oxidase activity, reactive oxygen species, and PAD4-dependent chromatin decondensation (55). At mucosal surfaces, NETs immobilize and neutralize microbes and viral particles, concentrate antimicrobial effectors, and shape subsequent antigen presentation and T-cell priming. Dysregulated or persistent NETs can conversely drive epithelial injury, fibrosis, thrombosis, and pro-tumor inflammation. Within the cervicovaginal niche, balanced NET formation may aid pathogen containment and epithelial repair, whereas excessive NETosis can sustain local damage and immunosuppression (56). In this context, vitamin D has been reported to promote efficient NET release while simultaneously limiting overproduction of pro-inflammatory cytokines, thereby supporting microbial control and barrier integrity without amplifying collateral tissue injury. Furthermore, vitD promotes the formation of NETs while concurrently limiting the release of pro-inflammatory cytokines (57). It also inhibits eosinophil recruitment through suppression of IL-15 and attenuates IgE-dependent mast cell degranulation. Collectively, these actions help maintain a balance between effective pathogen clearance and the preservation of tissue integrity.

#### 3.1.3 Vascular endothelium

VitD and its metabolites confer endothelial protection through both rapid and sustained mechanisms. In the non-genomic pathway, VitD_3_ binds membrane-bound VDR within seconds to minutes, activating AC/cAMP, IP3-DAG, and PI3K/Akt-Ser1177-eNOS signaling cascades (58). This elevates intracellular Ca^2+^, stimulates nitric oxide (NO) production, reinforces VE-cadherin-mediated adherens junctions, suppresses actin stress fiber formation, and reduces vascular permeability. Subsequently, 1,25(OH)_2_D enhances transcription of eNOS and heme oxygenase-1 (HO-1) via nuclear VDR, maintaining NO flux and counteracting oxidative stress (59). *In vivo* studies have confirmed that this dual action mitigates endothelial dysfunction and microvascular leakage in models of chronic kidney disease and sepsis.

#### 3.1.4 Intestinal epithelium and Paneth cells

In the gut, 1,25(OH)_2_D upregulates tight junction proteins such as ZO-1, Claudin-1, and Occludin, thereby restoring epithelial barrier integrity. It also enhances intracellular pattern recognition receptors (PRRs) like NOD2 and NLRP6, facilitating early detection of invading pathogens (60). Upon stimulation, intestinal epithelial cells, Paneth cells, and intraepithelial lymphocytes secrete antimicrobial peptides including LL-37, REG3γ, and α-defensin HD-5 (61). These peptides restrict microbial translocation and promote host–microbiota homeostasis, reducing the risk of chronic low-grade inflammation and metabolic disturbances.

Through the central TLR–CYP27B1–VDR axis, VitD orchestrates an integrated innate immune network encompassing localized antimicrobial responses, immune tolerance, vascular stabilization, and epithelial defense. Maintaining serum 25(OH)D levels within the optimal range of 50–75 nmol/L ensures a balance between anti-infective immunity and calcium safety. From a translational perspective, the development of selective VDR partial agonists and tissue-specific CYP27B1 activators holds promise for adjunctive therapies targeting tuberculosis, multidrug-resistant infections, and immune-mediated diseases (62).

### 3.2 Immunomodulatory roles of VitD in adaptive immunity

Activated T and B lymphocytes not only express the VDR but also induce the expression of CYP27B1, enabling the local conversion of circulating 25-hydroxyVitD [25(OH)D] into the bioactive form 1,25-dihydroxyVitD [1,25(OH)_2_D] within the lymphoid microenvironment. This establishes an autocrine/paracrine hormonal loop that allows VitD to reshape adaptive immune responses through multiple regulatory layers.

#### 3.2.1 T cells

1,25(OH)_2_D exerts lineage-specific effects on CD4^+^ T cells. *In vitro* studies demonstrate that it suppresses the transcription and secretion of Th1 cytokines, while enhancing IL-4 expression, thereby inhibiting Th1 differentiation and favoring Th2 polarization (63). In murine models of Crohn's disease, administration of 1,25(OH)_2_D, either alone or in combination with dexamethasone, further attenuates Th17 responses and increases Th2-associated markers (64). These findings suggest a potential role for vitamin D in mitigating mucosal inflammation through the suppression of IL-17.

Simultaneously, under the influence of TGF-β and IL-2, 1,25(OH)_2_D induces FoxP3 expression in naïve CD4^+^ T cells, promoting their differentiation into Tregs, and enhances IL-10 production by CD4^+^/CD25^+^ Tregs. This “Th1/Th17 suppression–Th2/Treg promotion” axis underpins VitD's potential protective role in Th1/Th17-driven autoimmune diseases (65, 66). Transcriptomic analyses of bronchoalveolar lavage fluid from COVID-19 patients further support this concept, revealing that Th1 cells locally activate the VitD pathway to suppress IFN-γ and upregulate IL-10, facilitating resolution of hyperinflammation (67).

CD8^+^CTLs are similarly regulated through VDR signaling. Upon infection or polyclonal activation, CTLs upregulate VDR expression, enabling 1,25(OH)_2_D to restrain their excessive proliferation by downregulating IL-2 and IL-12 signaling (68). This effect contributes to the restoration of the CD4/CD8 ratio, which serves as a surrogate marker for balanced immune activation. Clinical studies have reported that supplementation with 5,000–10,000 IU per day of vitamin D3 increases the CD4/CD8 ratio, reflecting a reduction in systemic immune activation.

#### 3.2.2 B cells

Resting B cells express negligible levels of VDR, but upon mitogen or antigen stimulation, both VDR and CYP27B1 are rapidly induced. 1,25(OH)_2_D directly inhibits B cell differentiation into antibody-secreting plasma cells, promotes apoptosis in activated B cells and plasmablasts, and markedly suppresses immunoglobulin and autoantibody production (69). Furthermore, VitD induces interleukin-10 (IL-10) expression in B cells and upregulates C–C chemokine receptor 10 (CCR10), promoting their differentiation toward an anti-inflammatory phenotype. This mechanism is particularly relevant in antibody-mediated autoimmune disorders.

It is noteworthy that multiple cross-sectional and retrospective studies have reported significantly reduced serum 25(OH)D levels in patients with chronic inflammation or autoimmune diseases. However, systemic inflammation itself may acutely deplete or redistribute VitD metabolites, as evidenced by human endotoxemia models showing a rapid drop in 25(OH)D levels within hours (70). Thus, hypovitaminosis D may represent both a contributing factor and a downstream effect of inflammation, necessitating careful differentiation in longitudinal interventional studies.

Through establishing a self-contained “25(OH)D–CYP27B1–1,25(OH)_2_D–VDR” loop within activated lymphocytes, VitD enables precise control over T/B cell proliferation, differentiation, and cytokine expression networks. This culminates in a coordinated program characterized by anti-inflammatory effects, immune tolerance promotion, and suppression of pathogenic antibody responses, offering a viable therapeutic strategy for various autoimmune and inflammatory diseases.

## 4 The role of VitD in different stages of cervical lesions

VitD exerts significant influence across various stages in the progression of cervical lesions from benign to malignant, although its primary functions differ at each stage (Table 1).

**Table 1:** Role of vitamin D across cervical lesion stages: mechanisms, evidence, and clinical implications.

**Stage**	**Clinical features**	**Primary VitD mechanisms**	**Clinical implications**	**Limitations**	**Ref**.
Polyp stage	Benign proliferative lesions, often driven by chronic inflammation; typically curable by physical removal	Anti-inflammatory and antimicrobial actions: strengthens mucosal barrier, upregulates antimicrobial peptides, potentially lowers chronic cervicitis and recurrence	Primarily primary prevention: maintaining adequate VitD may reduce formation/recurrence and secondary infection risk	Inference rather than direct evidence; never a substitute for procedural management	(71)
CIN1	Usually transient HPV infection; >60% regress within 1–2 years	Enhances antiviral mucosal immunity (innate responses, antimicrobial peptides), decreases local inflammation/oxidative stress, promotes epithelial repair	Assess and correct VitD deficiency as an adjunct to CIN1 management to promote HPV clearance and histologic regression	Thresholds/dosing not standardized; study heterogeneity; does not replace routine follow-up/management	(72)
CIN2/3	High-grade precancerous lesions; persistent high-risk HPV; LEEP/conization is standard of care	Pro-differentiation, anti-proliferative; improves inflammatory/metabolic milieu but unlikely to reverse established HSIL alone	Maintain adequate VitD alongside standard therapy to possibly slow progression, aid post-operative healing, and support HPV clearance; may help during conservative management of CIN2 in selected young patients	More immunosuppressive TIME; monotherapy effect limited; high-quality randomized evidence lacking	(73, 74)
Invasive cervical cancer	Deeply immunosuppressive TIME, established immune evasion; multimodal therapy required	*In vitro*: induces p21 and cell-cycle arrest; inhibits E6/E7 functions; may enhance radio-/immuno-therapy sensitivity; correcting deficiency supports systemic immunity and bone health	Supportive/sensitizing role: correct deficiency, potential radiosensitization and mitigation of immunosuppression; improves overall nutritional/bone status	Evidence largely *in vitro*/animal or retrospective; lacks phase III clinical proof; not a curative modality	(75, 76)

### 4.1 Polyp stage

Cervical polyps are benign proliferative lesions usually caused by chronic inflammatory stimulation. Although direct studies evaluating the effects of VitD on cervical polyps are lacking, it is hypothesized that sufficient levels of VitD may reduce the risk of polyp formation via its anti-inflammatory and antimicrobial properties. For example, VitD enhances mucosal immune barriers and increases antimicrobial peptide expression, which may reduce the incidence of chronic cervicitis and thereby indirectly decrease the risk of polyp development (71). Moreover, its immunomodulatory effects help maintain a balanced local immune response, preventing excessive inflammation that may otherwise induce tissue overgrowth. Therefore, maintaining adequate VitD levels through sunlight exposure or diet might help reduce the risk of benign cervical lesions, particularly in women with chronic inflammation. However, since cervical polyps can usually be resolved through physical removal, the role of VitD in this stage is likely preventive, such as reducing recurrence or secondary infections.

### 4.2 CIN1 stage

CIN1 is typically associated with transient HPV infection, and over 60% of cases regress spontaneously within 1–2 years. Multiple studies suggest that adequate VitD levels favor the regression of CIN1 and the clearance of HPV. A systematic review indicated that nearly half of the observational studies found lower VitD levels among HPV-infected women, and VitD deficiency was significantly associated with persistent high-risk HPV infection. A case–control study by Ozgu et al. further suggested that insufficient levels of VitD metabolites might contribute to persistent HPV DNA and CIN occurrence (*P* = 0.009). Interventional evidence also supports the benefit of supplementation. In a randomized controlled trial in Iran, CIN1 patients were given 50,000 IU of VitD3 every 2 weeks for 6 months. Results showed that 84.6% of patients in the VitD group experienced lesion regression, compared with 53.8% in the placebo group (*P* = 0.01) (72). Serum 25(OH)D levels significantly increased in the treatment group, along with improved fasting insulin and antioxidant markers. These findings indicate that VitD supplementation not only promotes histological regression of CIN1 but also improves metabolic and inflammatory profiles. Mechanistically, VitD may enhance mucosal antiviral immunity and reduce local inflammation and oxidative stress, thereby facilitating epithelial repair. In summary, VitD appears to play a protective role in the CIN1 stage by inhibiting lesion progression and promoting regression. Evaluating and correcting VitD deficiency could thus serve as a useful adjunct in CIN1 management to enhance natural viral clearance and lesion reversal.

### 4.3 CIN2/3 stage

CIN2 and CIN3 are precancerous lesions of the cervix, often indicating persistent high-risk HPV infection and a greater tendency toward neoplastic transformation. Surgery (e.g., LEEP or conization) remains the standard treatment. However, the immune microenvironment still plays a critical role in determining disease progression, and some CIN2 cases can regress spontaneously. The role of VitD in this stage is more complex. On the one hand, its ability to promote cell differentiation and inhibit proliferation may suppress lesion advancement; on the other hand, the microenvironment becomes increasingly immunosuppressive, making VitD alone insufficient for complete reversal. Clinical trials show that high-dose VitD supplementation for 6 months improves metabolic and inflammatory indices in CIN2/3 patients but has limited impact on lesion outcomes. As Avila et al. reported in a review, VitD supplementation did not significantly reduce recurrence rates during post-treatment follow-up in CIN2/3 patients. This suggests that VitD monotherapy is unlikely to eliminate established high-grade lesions (73). Nonetheless, vitamin D may provide auxiliary benefits by improving the local tissue microenvironment, such as reducing the risk of residual disease following surgical intervention and promoting HPV clearance. Some cohort studies also suggest that sufficient VitD status is associated with a lower risk of HSIL. In cases of conservative management of CIN2 (e.g., in young women avoiding immediate surgery), raising VitD levels may help promote lesion regression, though most supporting evidence stems from CIN1 studies. Therefore, although the effect of VitD diminishes in the CIN2/3 stage compared to earlier stages, it still holds potential in slowing lesion progression (74). Clinically, maintaining sufficient VitD levels alongside standard therapies may support immune function and tissue repair, though VitD should not be considered a replacement for excisional procedures.

### 4.4 Invasive cervical cancer stage

Once invasive cervical cancer has developed, the tumor microenvironment often becomes deeply immunosuppressive, characterized by well-established immune evasion mechanisms. At this stage, the antitumor effects of vitD are limited. According to Avila et al., although sufficient vitamin D levels may help prevent or regress early intraepithelial neoplasia, they confer minimal benefit in advanced cervical cancer, even when combined with chemotherapy (75). This may be due to tumor-intrinsic resistance mechanisms, such as dysregulated VDR signaling, and the presence of a stably TIME.

Nevertheless, VitD may still offer supportive benefits for cervical cancer patients. Firstly, VitD deficiency is common among these patients, and supplementation can improve systemic immune function and bone metabolism. Retrospective data show impaired immunity and elevated inflammatory markers in cervical cancer patients, which may be linked to low VitD levels (76). Secondly, *in vitro* studies have shown that VitD can exert antiproliferative effects on cervical cancer cell lines, such as inducing p21 expression to cause cell cycle arrest and inhibiting E6/E7 oncogenic functions (77). Preclinical models also suggest that VitD or its analogs can enhance tumor sensitivity to treatment. Notably, vitD may exert synergistic effects when combined with other therapies in advanced disease stages, such as enhancing radiosensitivity or alleviating immunosuppression during immunotherapy. Therefore, although VitD has a weaker impact in established cervical cancer than in precancerous stages, it remains a meaningful component of multimodal management.

## 5 The functions of VitD in the cervical-cancer immune microenvironment

**Figure 2:**
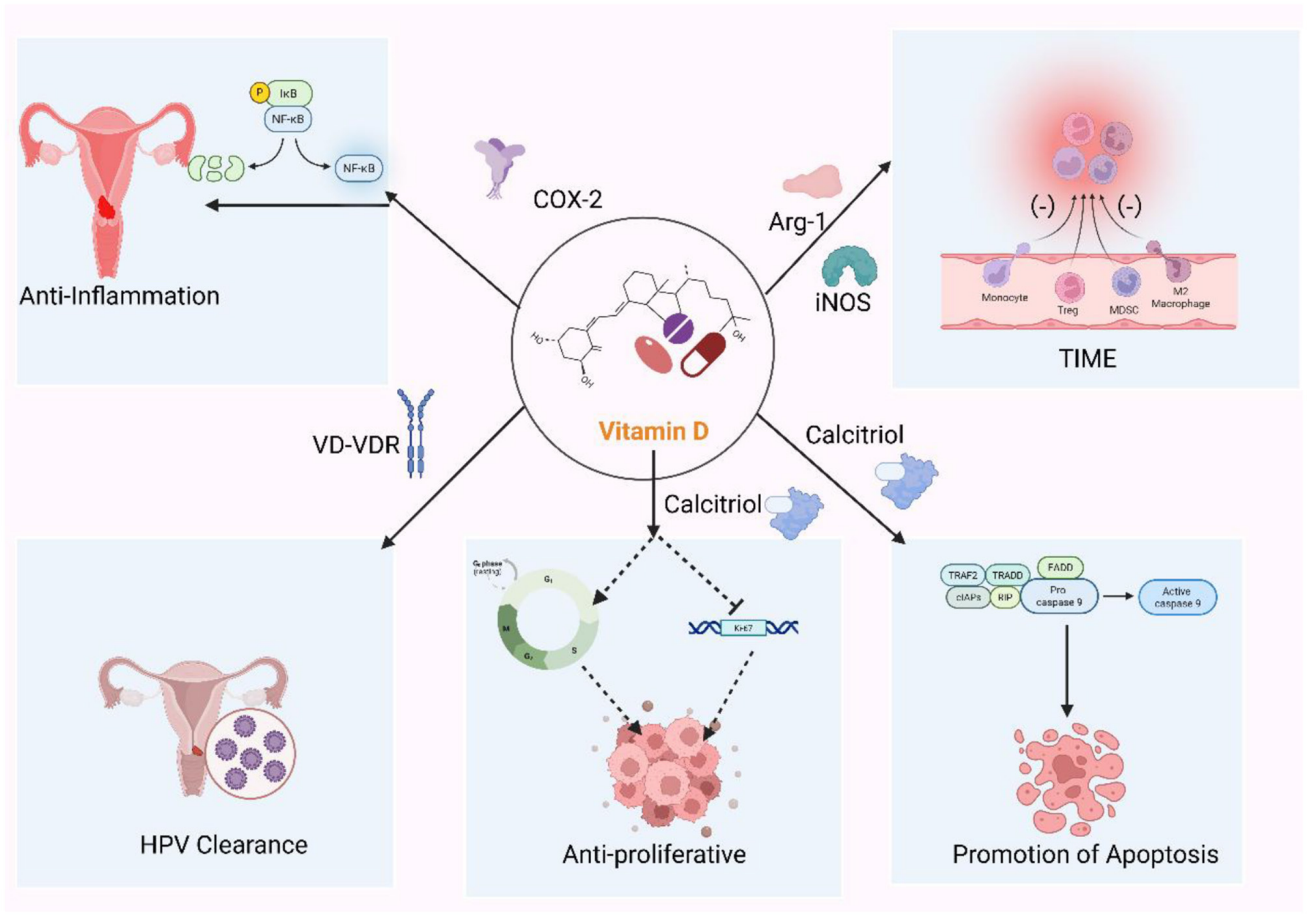
The role of vitamin D in cervical cancer. Vitamin D (VitD) coordinates antiviral and antitumor defenses across cervical disease. Via vitamin D receptor (VDR) signaling, it induces antimicrobial peptides (cathelicidin, β-defensins) and helps maintain a Lactobacillus-dominant microbiota, promoting clearance of high-risk human papillomavirus (HPV). VitD restrains tumor growth by up-regulating p21 and repressing HPV E6/E7, lowers the threshold for apoptosis (mitochondrial and Fas–caspase routes), and dampens COX-2/NF-κB–driven inflammation. It also remodels immunity by strengthening epithelial barrier function, tempering dendritic-cell activation, shifting CD4^+^ T-cell polarization toward a more regulatory profile, and reducing myeloid-derived suppression. These effects are strongest in infection and low-grade lesions (CIN1) and become adjunctive in HSIL and invasive cancer.

VitD acts as an “immune-microenvironment modulator”, reshaping local immunity through multiple, stage-specific mechanisms. Below, its principal actions are summarized by functional category (Figure 2).

### 5.1 Induction of antimicrobial/antiviral peptides and HPV clearance

One of the most classical immune effects of VitD is the induction of antimicrobial peptides, a feature of particular relevance in cervical pathology, where persistent high-risk HPV infection is the pivotal oncogenic driver. Epidemiological data support this link: in the US NHANES cohort, every 10 ng mL^−1^ decrease in serum 25-hydroxy-VitD [25(OH)D] was associated with a 14 % increase in high-risk HPV prevalence (OR = 1.14, 95 % CI 1.02–1.27). A Turkish case–control study similarly reported significantly lower VitD metabolites in HPV-positive women (*P* = 0.009) (78). In cervical epithelial cells, VD/VDR signaling markedly up-regulates cathelicidin and human β-defensin, peptides that not only exert direct virucidal activity but also recruit immune cells and accelerate mucosal healing. Avila and co-workers proposed that VitD-driven cathelicidin limits HPV spread and oncogenicity within the cervix. *In vitro*, VitD-treated epithelial cells display reduced susceptibility to HPV, in parallel with increased peptide expression (79). By stabilizing a Lactobacillus-dominant microbiota and neutralizing bacterial toxins, VitD may additionally lower the micro-inflammatory milieu that favors HPV persistence. Clinical observations in HIV-infected women further show that VitD supplementation shortens the duration of high-risk HPV carriage (80). Collectively, adequate VitD status appears to shift HPV infection from “persistent” to “transient”, explaining, at least in part, the lower cervical-cancer incidence in regions with abundant sunlight exposure.

### 5.2 Anti-proliferative and pro-differentiation effects

VD/VDR signaling suppresses cervical-cancer cell growth through coordinated inhibition of the cell cycle and down-regulation of HPV oncogenes. In HeLa cells, calcitriol up-regulates the cyclin-dependent-kinase inhibitor p21^CIP1/WAF1^, blocks Cyclin-E/CDK2 activity and arrests cells in the G0/G1 phase, which is corroborated by CCK-8 quantification and flow cytometry (81). In contrast, treatment with cholecalciferol or 25(OH)D3 in SiHa and CaSki cells does not affect Ki-67 expression or the G1-phase fraction. However, it significantly increases the sub-G1 population, which is indicative of DNA fragmentation and early apoptosis rather than classical G1 cell cycle arrest (82). These discrepancies reveal the distinct efficacy of systemic (hepatic–renal) vs. intracrine (CYP27B1-mediated) activation pathways, and underscore heterogeneity in hydroxylase/VDR expression among HPV-positive cell lines. Calcitriol further down-regulates HPV-E6/E7, preventing p53 degradation and thereby dismantling the viral proliferative programme (83, 84). Overall, VitD weakens cervical-cancer proliferation through a synergistic “G0/G1 blockade + oncogene repression” mechanism.

### 5.3 Promotion of apoptosis

VD/VDR concurrently engages the intrinsic (mitochondrial) and extrinsic (death-receptor) apoptotic cascades, forming a dual-pathway amplifier. Calcitriol triggers cytochrome-c release, apoptosome assembly and sequential activation of caspase-9 and caspase-3; in parallel, VDR lowers anti-apoptotic Bcl-2 and raises pro-apoptotic Bax, heightening mitochondrial permeability (85). Extrinsically, VDR transcriptionally induces Fas/Fas-ligand, activating caspase-8 and converging with the intrinsic pathway at caspase-3. This cooperation dramatically lowers the apoptotic threshold in HPV-driven or genomically stressed cells (86). In VDR-null C33A cells, calcitriol fails to induce CYP24A1 or initiate either cascade, confirming VDR dependence. Hence, VitD restrains cervical-cancer progression through combined growth arrest and apoptotic priming.

### 5.4 Dampening of pro-tumor inflammation

Persistent inflammation is a double-edged sword in carcinogenesis. VitD attenuates key inflammatory axes by down-regulating COX-2, thereby reducing pro-tumorigenic prostaglandin-E_2_, and by inhibiting chronic NF-κB activation (87). These effects are crucial in HPV infection, where viral oncoproteins (e.g., E6/E7) induce cytokine release that, in turn, sustains viral persistence. VitD interrupts this positive feedback loop, and epidemiological studies consistently show lower rates of persistent HPV and CIN progression in vitamin-D-replete individuals (88).

### 5.5 Modulation of immune cell compartments and immunometabolism

**Figure 3:**
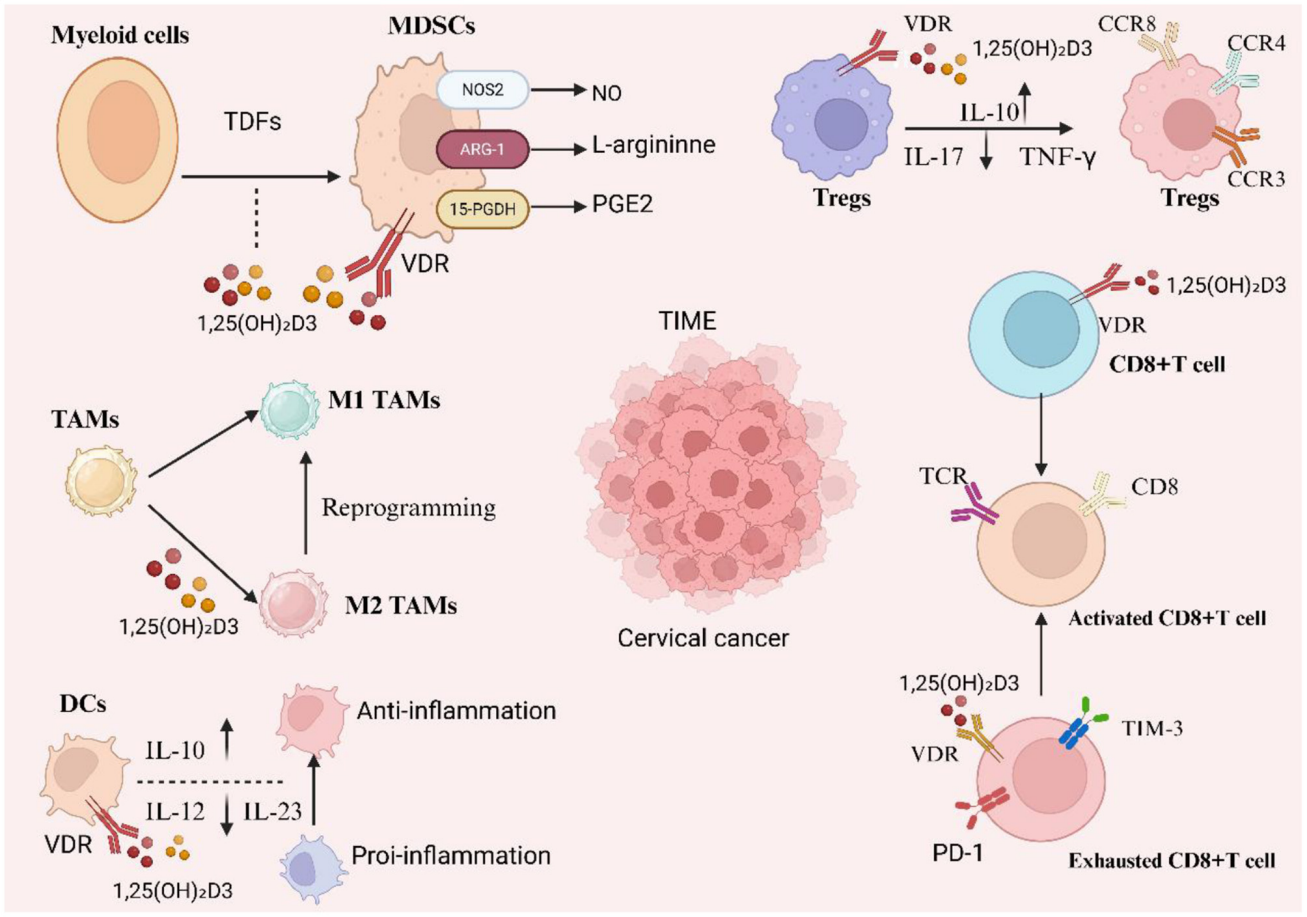
Vitamin D–VDR reprograms the cervical tumor immune microenvironment (TIME). 1,25(OH)_2_D3 acting via vitamin D receptor (VDR) counteracts tumor-derived factor–driven myelopoiesis by restraining MDSC suppressive programs (↓NOS2/NO and ARG-1–mediated L-arginine depletion; ↑15-PGDH to catabolize PGE_2_), promotes dendritic cells (DCs) with an anti-inflammatory profile (IL-10↑, IL-12/IL-23↓), and repolarizes tumor-associated macrophages toward M1 while limiting M2 states. In the lymphoid compartment, it enhances regulatory tuning (Tregs with IL-10↑ and IL-17/TNF-γ↓ and characteristic CCR3/CCR4/CCR8 patterns) and modulates CD8^+^ T cells—supporting activation through TCR signaling and potentially alleviating exhaustion (PD-1/TIM-3). Collectively, these actions soften chronic inflammation and reduce immune suppression within cervical cancer TIME.

VitD tailors DC maturation toward a tolerogenic phenotype. In early HPV infection or low-grade lesions, such semi-mature DCs curb injurious inflammation and favor a balanced microenvironment. In established tumors, however, excessive tolerogenic DCs may assist immune escape, implying a stage-specific benefit/risk profile. VD/VDR skews CD4^+^ T-cell polarization away from Th1/Th17 toward Th2/Treg lineages: this diminishes cytotoxic inflammation yet alleviates HPV-driven chronic damage (89). VitD-induced Tregs, though potentially suppressive in overt tumors, can prevent premalignant inflammation. Meanwhile, VD/VDR signaling limits the suppressive capacity of myeloid-derived suppressor cells (MDSCs) by down-regulating Arg-1 and iNOS, partly counterbalancing Treg expansion (90, 91). On the metabolic front, VitD reprogrammes immune-cell energy use, promoting oxidative phosphorylation in DCs and enhancing their IL-10 profile. It lowers reactive-oxygen species, thereby reducing DNA damage in chronically infected epithelia, and may shift tumor-associated macrophages toward an M1-like phenotype (92, 93). Together, these actions portray VitD as a metabolic and immunological nexus that maintains equilibrium between inflammation and tolerance throughout cervical-carcinogenesis continuum (Figure 3).

## 6 VitD-based therapeutic strategies and future perspectives

Given the multifaceted roles of VitD in immunity and the TME, a range of VitD-related therapeutic strategies have recently emerged for the prevention and treatment of cervical lesions.

### 6.1 Systemic supplementation of VitD

Systemic supplementation, administered either orally or intramuscularly, represents the most straightforward intervention for individuals with VitD deficiency. This strategy is particularly promising for the prevention and conservative management of low-grade cervical lesions (94). As discussed earlier, randomized controlled trials have shown that high-dose VitD supplementation significantly increases the spontaneous regression rate of CIN1. This suggests that, in clinical settings, oral high-dose VitD could be considered as an adjunctive therapy for CIN1 patients with documented deficiency. Similarly, for women with persistent HPV infection but no progression to high-grade lesions, maintaining adequate VitD levels (e.g., serum 25(OH)D > 30 ng/mL) may facilitate viral clearance and reduce the risk of disease advancement (95). This approach is cost-effective and generally safe; toxicity is rare if levels are maintained within the physiological range. Moreover, systemic VitD supplementation may enhance overall immune responsiveness. One study found that cancer patients undergoing immunotherapy experienced significantly better outcomes when their VitD levels were sufficient. While VitD is not a conventional anticancer agent, it functions as a foundational immune-supportive therapy and should be integrated into the comprehensive management of cervical lesions (96).

### 6.2 Local application of VitD

Given that the cervix and vagina are accessible for direct drug delivery, several studies have explored local administration of VitD or its derivatives. Vaginal suppositories or gels containing active VitD compounds could potentially deliver high concentrations directly to the lesion site, thereby minimizing systemic side effects and enhancing localized immune responses (97). While studies in this area are limited, dermatological applications have yielded promising results. For instance, topical use of VitD analogs such as calcipotriol has demonstrated excellent efficacy in treating HPV-related cutaneous warts, achieving complete clearance in 73% of cases compared to negligible effects in placebo groups. As cervical lesions are also driven by HPV, it is reasonable to hypothesize that localized VitD therapy could be effective (98, 99). Innovative formulations such as nano-gel systems delivering calcitriol intravaginally may enhance epithelial targeting, promote viral clearance, induce apoptosis in dysplastic cells, and minimize hypercalcemia. Preliminary studies have also explored intralesional VitD injection for treating genital warts and CIN, though data remain scarce. Advantages of local therapy include rapid onset, minimal off-target toxicity, and potential for repeated administration (100). However, challenges such as compound instability and unknown effects on mucosal tissues with long-term use remain. Small-scale clinical trials are warranted to evaluate the efficacy of localized VitD treatment in CIN. If validated, this could offer a conservative option for CIN2/3 and a potential adjuvant for residual disease management.

VitD deficiency is common because only a small fraction of the recommended daily intake can be obtained even from the richest dietary sources such as fatty fish, fish oil, and egg yolks. Supplementation is therefore important to maintain serum 25-hydroxyvitamin D [25(OH)D] at approximately 30 ng/mL (75 nmol/L) for bone health and at 40–60 ng/mL (100–150 nmol/L) for lower cardiovascular and all-cause mortality risks (101). In normal-weight adults, these targets can typically be achieved with 2,000–5,000 IU/day, whereas individuals with obesity may require up to 10,000 IU/day because responses vary with genetics, body weight, health status, and medications (102). Higher-dose intermittent cholecalciferol regimens, such as 50,000 IU weekly or 100,000 IU monthly, have been shown to maintain 25(OH)D concentrations of 40–60 ng/mL without signs of vitamin D toxicity; toxicity is rare relative to other fat-soluble vitamins (103). When employing higher doses, periodic monitoring of 25(OH)D and serum calcium is advisable to ensure safety and goal attainment.

### 6.3 VDR agonists and VitD analogs

Calcitriol, the natural ligand for the VDR, has a narrow therapeutic window and may cause hypercalcemia at high doses. Hence, the development of VitD analogs with selective VDR agonism and reduced calcium-related activity has become a strategic priority. Calcipotriol, a synthetic VitD analog used in psoriasis, modulates keratinocyte proliferation and inflammation and has demonstrated efficacy in clearing HPV-related cutaneous warts (104). In the context of cervical disease, next-generation VDR agonists, including third-generation analogs with minimal calcemic activity, hold potential therapeutic value. *In vitro* studies have shown that certain analogs (e.g., EB1089) exhibit antiproliferative and pro-apoptotic effects on cervical cancer cells, with comparable or superior potency to calcitriol and lower toxicity (105). Paricalcitol, a selective intravenous VDR agonist primarily used for secondary hyperparathyroidism in chronic kidney disease, has also been investigated for its anti-inflammatory properties in cancer supportive care (106). It may mitigate radiation-induced tissue inflammation during chemoradiotherapy in cervical cancer patients. Overall, novel VDR-targeted agents hold promise as pharmaceutical interventions for cervical neoplasia, pending further validation of safe dosage ranges and clinical efficacy.

### 6.4 VitD as a radiosensitizer

Radiotherapy remains a cornerstone of cervical cancer treatment, yet tumor radioresistance limits therapeutic efficacy. Emerging evidence suggests that VitD can act as a radiosensitizer (107). Zhang et al. reported that pretreatment with VitD significantly sensitized VDR-positive cervical cancer cells to ionizing radiation by promoting radiation-induced apoptosis (108). Mechanistically, VitD downregulated the autophagy regulator Ambra1, impairing tumor cell autophagic responses that would otherwise protect against radiation-induced damage. Notably, VitD did not enhance DNA repair but rather shifted the balance toward cell death pathways (109). Animal studies support this synergistic interaction: combined VitD and radiotherapy more effectively suppressed tumor growth than radiotherapy alone. Thus, in VDR-expressing cervical tumors, VitD or calcitriol supplementation during radiotherapy may improve treatment outcomes. Given its ease of implementation, VitD supplementation around the time of radiotherapy represents a feasible clinical strategy (110). However, electrolyte disturbances due to VitD overdose must be monitored, and optimal dosage and timing remain to be established. Available data justify further investigation of VitD as a low-toxicity radiosensitizer to enhance local tumor control.

### 6.5 VitD combined with immunotherapy

Immune checkpoint inhibitors (ICIs), such as anti–PD-1/PD-L1 antibodies, have shown efficacy in subsets of patients with advanced cervical cancer, but overall response rates remain limited. VitD has potential to improve ICI responsiveness by modulating the immunosuppressive TME and enhancing T-cell functionality (111). Specifically, VitD reduces PD-1 expression on T cells, thereby mitigating exhaustion signals. It also decreases the suppressive activities of MDSCs and Tregs, supporting a more robust effector T-cell response. Clinical studies support this mechanism: a prospective study in patients with advanced solid tumors found that those receiving concurrent VitD supplementation during ICI treatment had significantly improved progression-free survival and disease control rates compared to VitD-deficient patients (112). In melanoma, patients with sufficient VitD levels also demonstrated higher objective response rates. These findings highlight VitD as a promising immunotherapeutic adjuvant. In cervical cancer, trials evaluating agents such as pembrolizumab may benefit from stratification by VitD status or integration of VitD supplementation into treatment protocols. Furthermore, VitD may enhance the efficacy of therapeutic HPV vaccines by maintaining adequate immune function, facilitating stronger T-cell responses. Experts recommend maintaining serum 25(OH)D levels within the range of 30–50 ng/mL in patients undergoing immunotherapy to optimize immune support.

### 6.6 Other combined strategies

Additional VitD-based combination strategies include.

VitD and chemotherapy: While a study by Avila et al. reported limited efficacy for VitD combined with conventional chemotherapy in advanced cervical cancer, synergism may exist for select agents. For example, VitD analogs combined with cisplatin demonstrated enhanced growth inhibition in cervical cancer cell lines (113, 114).

VitD and targeted small molecules: VitD may complement agents targeting the PI3K/Akt pathway by attenuating proliferation via nuclear receptor signaling and overcoming resistance (115).

VitD and microbiota modulation: VitD influences both gut and vaginal microbiota. Co-administration with probiotics or microbial therapeutics may restore vaginal microbial balance in HPV-infected individuals (e.g., by increasing Lactobacillus dominance), enhancing antiviral immunity (116).

VitD and NSAIDs: Dual inhibition of the COX-2/PGE2 axis through VitD and non-steroidal anti-inflammatory drugs (NSAIDs) may yield additive anti-inflammatory and chemopreventive effects—an area worth exploring for cervical cancer prevention (117).

Finally, VitD contributes to improving cancer patient quality of life, We also summarized the current relevant clinical trials (Table 2). Cervical cancer patients frequently suffer from fatigue, muscle weakness, and bone loss due to treatment or disease burden. Adequate vitamin D supplementation may help mitigate these effects by supporting musculoskeletal health and overall vitality, thereby reinforcing its importance in comprehensive patient care.

**Table 2:** Clinical trials related to vitamin D deficiency in tumors.

**NCT number**	**Study title**	**Conditions**	**Interventions**	**Phases**
NCT03038516	‘Palliative-D' Vitamin D to Palliative Cancer Patients	Vitamin D Deficiency	DRUG: Cholecalciferol|DEVICE: Placebo	PHASE2
NCT03594214	Prognostic Value of Vitamin D Levels in Egyptian Females With Breast Cancer	Vitamin D Deficiency	DIAGNOSTIC_TEST: serum vitamin d level measured by ELISA kit	NA
NCT05506696	Vitamin D Supplementation Study	Colorectal Cancer|Vitamin D Deficiency	DIETARY_SUPPLEMENT: Fultium	NA
NCT03144128	Vitamin D for Muscle Metabolic Function in Cancer Cachexia	Cancer Cachexia|Vitamin D Deficiency	DIETARY_SUPPLEMENT: Vitamin D|DIETARY_SUPPLEMENT: Placebo	NA
NCT01817231	Epidemiological Analysis of Vitamin D and Breast Cancer Risk in Saudi Arabian Women	Breast Cancer|Vitamin D Deficiency		NA
NCT01575717	The Effect of Vitamin D Repletion in Patients With Hepatocellular Carcinoma on the Orthotopic Liver Transplant List	Vitamin D Deficiency|Hepatocellular Carcinoma	DRUG: Vitamin D3 4000 IU|DRUG: Vitamin D3 2000 IU	NA
NCT03986268	Vitamin D Can Increase Pathological Response of the Breast Cancer Patients Treated With Neoadjuvant Therapy	Vitamin D Deficiency|Chemotherapy Effect|Pathology	DRUG: Vit D	NA
NCT03584529	Association Between Vitamin D and the Development of Uterine Fibroids	Gynecological Disease|Vitamin D Deficiency	DRUG: Vitamin D 3	NA
NCT06551688	Influence of Preoperative Vitamin D Level on Postoperative Pain in Breast Cancer Surgery Patients	Cancer Breast|Hypovitaminosis D	DIAGNOSTIC_TEST: Serum 25(OH)D level	NA
NCT04621500	Vitamin D Supplementation in RNA-seq Profiles of Single-core Prostate Samples, Among Veterans	Prostate Cancer|Vitamin D Deficiency|Stress Reaction	DRUG: cholecalciferol|PROCEDURE: Standard of Care Prostate Biopsy - collection of 13th core for RNA-seq|PROCEDURE: Standard of Care Prostate Biopsies at baseline and after one year vitamin D3 supplementation|DIAGNOSTIC_TEST: Allostatic Load	PHASE2
NCT04030182	Vitamin D Deficit in Women With Uterine Fibroids	Leiomyoma|Vitamin D Deficiency|Fibroid Uterus	DIAGNOSTIC_TEST: Level of Vitamin D	NA
NCT05950204	Effect of Supplementation With 蠅-3 Fatty Acids, Vitamin D and Calcium in Patients With Acute Lymphoblastic Leukemia.	Acute Lymphoblastic Leukemia|Vitamin d Deficiency	DIETARY_SUPPLEMENT: 蠅-3 polyunsaturated fatty acids (DHA and EPA), Vitamin D (cholecalciferol), Calcium (calcium carbonate)	NA
NCT03472833	High-dose Vitamin D3 in Pancreas Cancer	Pancreas Cancer|Vitamin D Deficiency|Quality of Life	DRUG: High-dose|DRUG: Standard dose	PHASE3
NCT05650151	Peri-operative Vitamin D Therapy for Hepatectomy	Vitamin D Deficiency|Hepatocellular Carcinoma|Perioperative Complication	DIETARY_SUPPLEMENT: Vitamin D|DIETARY_SUPPLEMENT: Placebo	NA
NCT03871868	Vitamin D Levels And Myoma Uteri	Myoma;Uterus|Vitamin D Deficiency|Polycystic Ovary		NA

## 7 Limitations, safety, and implementation considerations of VitD-related therapeutic strategies

VitD-based strategies for cervical disease include systemic supplementation, cervicovaginal local delivery, selective VDR agonists, radiosensitization, and combinations with immunotherapy or other agents. Their translational promise coexists with several constraints.

First, the strength of evidence varies across indications. Randomized trials in deficient populations suggest that high-dose cholecalciferol can increase spontaneous regression of low-grade CIN. By contrast, support for high-grade CIN and invasive cancer derives largely from small, heterogeneous studies, preclinical models, and secondary analyses. Well-stratified phase II and real-world trials are needed that enroll by baseline 25-hydroxyvitamin D [25(OH)D], VDR expression, HPV genotype, and lesion grade, and that use endpoints such as histologic regression, durable HPV clearance, and quality of life rather than short-term surrogates.

Second, measurement and dosing introduce variability. Immunoassays and LC–MS for 25(OH)D are not interchangeable, and total concentrations may not reflect the bioavailable fraction, which depends on VitD–binding protein and genetic variation. Target ranges for cervical outcomes are not standardized, and responses may differ between daily and intermittent regimens. Overall safety is favorable, yet high-dose or intermittent schedules warrant attention to hypercalcemia, nephrolithiasis risk, and drug interactions. Thiazide diuretics, calcium supplements, digitalis, anticonvulsants, glucocorticoids, and antiretrovirals can modify vitamin D metabolism or amplify adverse effects; dose intensification should be accompanied by monitoring of 25(OH)D and serum calcium.

Third, tissue context sets an efficacy ceiling. VDR expression is heterogeneous across the cervical disease spectrum and may decline in advanced tumors, potentially attenuating the benefit of systemic VitD or VDR agonists. On-target activity also depends on intracrine activation via CYP27B1, the prevailing inflammatory tone, and the state of the local microbiome. In radiosensitization settings, the timing relative to fractionated radiotherapy and the balance between apoptosis promotion and autophagy suppression require prospective optimization to avoid off-target toxicity. For combinations with immune checkpoint inhibition, associations between VitD sufficiency and outcomes are hypothesis-generating; prospective studies should incorporate immune phenotyping, including PD-L1, T-cell exhaustion states, and the neutrophil-to-lymphocyte ratio, to identify subgroups most likely to benefit.

Fourth, delivery and formulation pose practical barriers. Cervicovaginal gels, rings, and nanocarriers can concentrate drug at lesion sites while limiting systemic exposure, but stability in the vaginal milieu, mucosal retention, patient acceptability, and the regulatory pathway for device–drug combinations must be addressed. Although selective VDR agonists offer a wider therapeutic window than calcitriol, dose-finding that preserves antitumor and immunomodulatory effects while minimizing calcemic activity remains a key translational step.

Fifth, implementation requires system-level solutions. Harmonized assays, shared data standards, and pooled procurement can reduce heterogeneity and cost. Adherence, access, and equity are central, particularly in high-HPV-burden, resource-limited settings. Future studies should use adaptive, biomarker-enriched designs; include cervicovaginal pharmacokinetics and pharmacodynamics; and embed mechanistic readouts such as LL-37 induction, antigen-presentation transcripts, and spatial immune metrics. These steps will clarify where vitamin D functions primarily as prevention or immune support, where it acts as a true therapeutic adjunct, and where alternative strategies should be prioritized.

## 8 Conclusion

The future of cervical cancer prevention and treatment will increasingly rely on multidisciplinary collaboration and integrated management strategies. VitD, owing to its unique immunomodulatory and antitumor properties, holds promise as an effective adjunctive intervention for cervical lesions. From a public health perspective, improving VitD nutritional status across populations may serve as a feasible strategy to reduce the risk of persistent HPV infection and the development of precancerous cervical lesions. In clinical practice, targeted VitD supplementation for high-risk individuals (such as those with persistent HPV infection) may help delay or prevent the onset and progression of CIN. For patients already diagnosed with cervical lesions, incorporating VitD into therapeutic regimens may enhance treatment response, improve prognosis, and contribute to better quality of life.

However, several key scientific questions remain unresolved. For instance, the dose–response relationship of VitD in cervical disease is still unclear. The impact of VDR gene polymorphisms on treatment efficacy also warrants further investigation, as certain VDR genotypes have been associated with increased susceptibility to cervical cancer. In addition, how VitD supplementation could be synergistically integrated with existing preventive strategies, such as HPV vaccination, is an important area for future research. Given that VitD acts as a metabolic and immune regulator with typically slow and cumulative effects, its clinical benefits may not be immediately apparent. Therefore, outcome evaluation should emphasize long-term follow-up indicators, such as lesion progression, recurrence rates, and patient-reported quality of life, rather than relying solely on short-term histopathological changes.

In conclusion, the dynamic interplay between VitD, the immune microenvironment, and cervical disease progression is offering new insights into the pathogenesis and management of cervical cancer. VitD appears to exert influence at multiple critical stages of cervical carcinogenesis. With continued mechanistic investigations and high-quality clinical trials, this safe and naturally occurring molecule has the potential to be fully harnessed to develop optimized strategies, ultimately contributing to the global reduction of cervical cancer burden and improving patient outcomes.

## References

[1] Voelker RA. Cervical cancer screening. JAMA. (2023) 330:2030. 10.1001/jama.2023.21987 37889510

[2] Francoeur AA Monk BJ Tewari KS. Treatment advances across the cervical cancer spectrum. Nat Rev Clin Oncol. (2025) 22:182–99. 10.1038/s41571-024-00977-w 39753753

[3] Lycke KD Kahlert J Petersen LK Damgaard RK Cheung LC Gravitt PE. Untreated cervical intraepithelial neoplasia grade 2 and subsequent risk of cervical cancer: population based cohort study. BMJ. (2023) 383:e075925. 10.1136/bmj-2023-075925 38030154 PMC10685285

[4] Wang J Elfström KM Dillner J. Human papillomavirus-based cervical screening and long-term cervical cancer risk: a randomised health-care policy trial in Sweden. Lancet Public Health. (2024) 9:e886–95. 10.1016/S2468-2667(24)00218-4 39486904

[5] Garg P Krishna M Subbalakshmi AR Ramisetty S Mohanty A Kulkarni P. Emerging biomarkers and molecular targets for precision medicine in cervical cancer. Biochimica et biophysica acta Reviews on cancer. (2024) 1879:189106. 10.1016/j.bbcan.2024.189106 38701936

[6] Duska LR Podwika SE Randall LM. Top advances of the year: cervical cancer. Cancer. (2024) 130:2571–6. 10.1002/cncr.35334 38651760

[7] How JA Jazaeri AA. Immunotherapy in locally advanced cervical cancer: integrating KEYNOTE-A18 into management strategies. Med. (2024) 5:487–9. 10.1016/j.medj.2024.05.001 38878765 PMC12004455

[8] Guo W Dai L Qiu L. T cell subsets in cervical cancer tumor microenvironment: advances and therapeutic opportunities. Front Immunol. (2025) 16:1612032. 10.3389/fimmu.2025.1612032 40539072 PMC12176900

[9] Liu C Li X Huang Q Zhang M Lei T Wang F. Single-cell RNA-sequencing reveals radiochemotherapy-induced innate immune activation and MHC-II upregulation in cervical cancer. Signal Transduct Target Ther. (2023) 8:44. 10.1038/s41392-022-01264-9 36710358 PMC9884664

[10] Gutiérrez-Hoya A. Soto-Cruz I. NK cell regulation in cervical cancer and strategies for immunotherapy. Cells. (2021) 10:3104. 10.3390/cells10113104 34831327 PMC8619016

[11] Daryabor G Gholijani N Kahmini Kahmini FR: a review of the critical role of vitamin D axis on the immune system. Exp Mol Pathol. (2023) 132–133:104866. 10.1016/j.yexmp.2023.104866 37572961

[12] Giampazolias E Pereira da Costa M Lam KC Lim KHJ Cardoso A Piot C. Vitamin D regulates microbiome-dependent cancer immunity. Science. (2024) 384:428–37. 10.1126/science.adh7954 38662827 PMC7615937

[13] Ross TL Neale RE Na R Webb PM. Vitamin D status during and after treatment and ovarian cancer survival. Cancer Causes Control. (2024) 35:1–8. 10.1007/s10552-023-01757-0 37526780 PMC10764528

[14] Wimalawansa SJ: Vitamin D's Impact on cancer incidence and mortality: a systematic review. Nutrients. (2025) 17:2333. 10.3390/nu17142333 40732958 PMC12298439

[15] Zhang Y Xu Y Zhong W Zhao J Liu X Gao X. Vitamin D and immune checkpoint inhibitors in lung cancer: a synergistic approach to enhancing treatment efficacy. Int J Mol Sci. (2025) 26:4511. 10.3390/ijms26104511 40429656 PMC12111780

[16] Baker E MacDonald A Tennant S. Approach to cervical polyps in primary care. Can Fam Physician. (2025) 71:26–30. 10.46747/cfp.710126 39843194 PMC11753288

[17] Wakimoto T Hayashi S Koh I Yamamoto R Ishii K: Relationship between unremoved cervical polyp in pregnancy and spontaneous preterm birth. Am J Obstetr Gynecol. (2022) 227:899.e891–6. 10.1016/j.ajog.2022.06.064 35841937

[18] Wang M Ye M Shen N Pan W Zhang H Wang X. Management of pregnant women with endocervical and decidual polyps: a systematic review and meta-analysis. Arch Gynecol Obstet. (2025) 312:375–84. 10.1007/s00404-025-08056-w 40407879 PMC12334433

[19] Chu WL Rajasekar T Ahamed M Ibraheim M Owen K. Conundrum in primary care: should all cervical polyps be removed? Br J Gener Pract. (2024) 74:254–5. 10.3399/bjgp24X737985 38902074

[20] Bruno MT Cassaro N Bica F Boemi S. Progression of CIN1/LSIL HPV persistent of the cervix: actual progression or CIN3 coexistence. Infect Dis Obstet Gynecol. (2021) 2021:6627531. 10.1155/2021/6627531 33776406 PMC7972837

[21] Gardella B Dominoni M Pasquali MF Melito C Fiandrino G Cesari S. Low-grade cervical intraepithelial neoplasia (CIN1) evolution: analysis of opportunistic preventive vaccination role. Vaccines. (2023) 11:284. 10.3390/vaccines11020284 36851162 PMC9961273

[22] Peronace C Cione E Abrego-Guandique DM Fazio M Panduri G Caroleo MC. FAM19A4 and hsa-miR124-2 double methylation as screening for ASC-H- and CIN1 HPV-positive women. Pathogens. (2024) 13:312. 10.3390/pathogens13040312 38668267 PMC11054986

[23] Major AL Skrivánek A Grandjean EM Dvorák V Malík T Pluta M. An Adsorptive and antioxidant vaginal gel clears high-risk HPV- and p16/Ki-67-associated abnormal cytological cervical findings: a *post-hoc* subgroup analysis of a prospective randomized controlled trial on CIN2 and p16 positive CIN1. Front Med. (2021) 8:645559. 10.3389/fmed.2021.645559 34113633 PMC8185015

[24] Li X Chen Y Xiong J Chen P Zhang D Li Q. Biomarkers differentiating regression from progression among untreated cervical intraepithelial neoplasia grade 2 lesions. J Adv Res. (2025) 74:391–402. 10.1016/j.jare.2024.09.009 39260797 PMC12302658

[25] Bukowski A Hoyo C Vielot NA Graff M Kosorok MR Brewster WR. Epigenome-wide methylation and progression to high-grade cervical intraepithelial neoplasia (CIN2+): a prospective cohort study in the United States. BMC Cancer. (2023) 23:1072. 10.1186/s12885-023-11518-6 37932662 PMC10629205

[26] Kechagias KS Kalliala I Bowden SJ Athanasiou A Paraskevaidi M Paraskevaidis E. Role of human papillomavirus (HPV) vaccination on HPV infection and recurrence of HPV related disease after local surgical treatment: systematic review and meta-analysis. BMJ. (2022) 378:e070135. 10.1136/bmj-2022-070135 35922074 PMC9347010

[27] Li ZY Wang K Shen XL Li Q. Factors associated with CIN2-3 recurrence: a single center retrospective analysis. Hum Vaccin Immunother. (2025) 21:2469410. 10.1080/21645515.2025.2469410 39982437 PMC11849945

[28] Wittenborn J Kupec T Iborra S Najjari L Kennes LN Stickeler E. CIN2 + detection in high-risk HPV patients with no or minor cervical cytologic abnormalities: a clinical approach validated by machine learning. Arch Gynecol Obstet. (2023) 307:881–90. 10.1007/s00404-023-06953-6 36780042 PMC9984503

[29] Dominoni M Inzani FS Gritti A Pasquali MF Mauri M Eldar A. The role of programmed death-ligand 1 (PDL-1) in high-grade cervical intraepithelial neoplasia (CIN2+) development and recurrence: a systematic review of literature about HPV-CIN2+-PDL-1 axis. Pathol Res Pract. (2024) 264:155712. 10.1016/j.prp.2024.155712 39522315

[30] Xu J Ji Q Kong Q Lv L Zhu B Huang X. Minimally invasive diagnosis of precancerous cervical lesions using single-cell peripheral immune atlas. Cell Rep Med. (2025) 6:102149. 10.1016/j.xcrm.2025.102149 40412381 PMC12208321

[31] Choi YJ Lee A Kim TJ Jin HT Seo YB Park JS. E2/E6 ratio and L1 immunoreactivity as biomarkers to determine HPV16-positive high-grade squamous intraepithelial lesions (CIN2 and 3) and cervical squamous cell carcinoma. J Gynecol Oncol. (2018) 29:e38. 10.3802/jgo.2018.29.e38 29400024 PMC5920222

[32] Zhong F Wang T Li W Zhang H Zeng X Geisler D. Associations of single vs. multiple human papillomavirus infections with the prevalence of cervical intraepithelial neoplasia 2/3 and squamous cell carcinoma lesions: human papillomavirus type-specific attribution. Lab Invest. (2024) 104:100328. 10.1016/j.labinv.2024.100328 38237737

[33] Andralojc KM Elmelik D Rasing M Pater B Siebers AG Bekkers R. Targeted RNA next generation sequencing analysis of cervical smears can predict the presence of hrHPV-induced cervical lesions. BMC Med. (2022) 20:206. 10.1186/s12916-022-02386-1 35676700 PMC9178797

[34] Zhang T Zhuang L Muaibati M Wang D Abasi A Tong Q. Identification of cervical cancer stem cells using single-cell transcriptomes of normal cervix, cervical premalignant lesions, and cervical cancer. EBioMedicine. (2023) 92:104612. 10.1016/j.ebiom.2023.104612 37224771 PMC10277926

[35] Yao S Zhao L Chen S Wang H Gao Y Shao NY Dai M. Cervical cancer immune infiltration microenvironment identification, construction of immune scores, assisting patient prognosis and immunotherapy. Front Immunol. (2023) 14:1135657. 10.3389/fimmu.2023.1135657 36969161 PMC10037308

[36] Li J Cao Y Liu Y Yu L Zhang Z Wang X. Multiomics profiling reveals the benefits of gamma-delta (γδ) T lymphocytes for improving the tumor microenvironment, immunotherapy efficacy and prognosis in cervical cancer. J Immunother Cancer. (2024) 12:e008355. 10.1136/jitc-2023-008355 38199610 PMC10806547

[37] Gao X Wang Q Huang T Xu C Yang X Zhang L. Cervical cancer-produced neuromedin-B reprograms Schwann cells to initiate perineural invasion. Cell Death Dis. (2024) 15:636. 10.1038/s41419-024-07030-9 39214988 PMC11364772

[38] Xia P Zhou J Shen R Wang D. Deciphering the cellular and molecular landscape of cervical cancer progression through single-cell and spatial transcriptomics. NPJ Precis Oncol. (2025) 9:158. 10.1038/s41698-025-00948-z 40437003 PMC12120119

[39] Lin Z Zhou Y Liu Z Nie W Cao H Li S. Deciphering the tumor immune microenvironment: single-cell and spatial transcriptomic insights into cervical cancer fibroblasts. J Exp Clin Cancer Res. (2025) 44:194. 10.1186/s13046-025-03432-5 40616092 PMC12228347

[40] Zhou R Xie Y Wang Z Liu Z Lu W Li X. Single-cell transcriptomic analysis reveals CD8 + T cell heterogeneity and identifies a prognostic signature in cervical cancer. BMC Cancer. (2025) 25:498. 10.1186/s12885-025-13901-x 40102789 PMC11916872

[41] Zhou L Liu J Yao P Liu X Chen F Chen Y. Spatial transcriptomics reveals unique metabolic profile and key oncogenic regulators of cervical squamous cell carcinoma. J Transl Med. (2024) 22:1163. 10.1186/s12967-024-06011-y 39741285 PMC11687147

[42] Li C Hua K. Dissecting the single-cell transcriptome network of immune environment underlying cervical premalignant lesion, cervical cancer and metastatic lymph nodes. Front Immunol. (2022) 13:897366. 10.3389/fimmu.2022.897366 35812401 PMC9263187

[43] Bergadà L Pallares J Maria Vittoria A Cardus A Santacana M Valls J. Role of local bioactivation of vitamin D by CYP27A1 and CYP2R1 in the control of cell growth in normal endometrium and endometrial carcinoma. Lab Invest. (2014) 94:608–22. 10.1038/labinvest.2014.57 24732451

[44] Yang JJ Fan HZ Tian T Wu MP Xie CN Huang P. Impact of CYP2R1, CYP27A1 and CYP27B1 genetic polymorphisms controlling vitamin D metabolism on susceptibility to hepatitis C virus infection in a high-risk Chinese population. Arch Virol. (2019) 164:2909–18. 10.1007/s00705-019-04378-8 31520221

[45] Huang J Liang Q Ye Y Lan Z Chen A Yan J. GDF11 alleviates vascular calcification in VitD(3)-overloaded mice through inhibition of inflammatory NF-κB signal. FASEB J. (2025) 39:e70677. 10.1096/fj.202500029R 40432427

[46] Nguyen-Huynh NT Osz J Peluso-Iltis C Rochel N Potier N Leize-Wagner E. Monitoring of the retinoic acid receptor-retinoid X receptor dimerization upon DNA binding by native mass spectrometry. Biophys Chem. (2016) 210:2–8. 10.1016/j.bpc.2015.10.006 26558701

[47] Ismailova A White JH. Vitamin D, infections and immunity. Rev Endocr Metab Disord. (2022) 23:265–77. 10.1007/s11154-021-09679-5 34322844 PMC8318777

[48] Yimamu Y Ohtani A Takei Y Furuichi A Kamei Y Yamanaka-Okumura H. 25-hydroxyvitamin D-1α-hydroxylase (CYP27B1) induces ectopic calcification. J Clin Biochem Nutr. (2022) 71:103–11. 10.3164/jcbn.22-16 36213783 PMC9519415

[49] Miao S Liu H Yang Q Zhang Y Chen T Chen S. Cathelicidin peptide LL-37: a multifunctional peptide involved in heart disease. Pharmacol Res. (2024) 210:107529. 10.1016/j.phrs.2024.107529 39615616

[50] Martens PJ Gysemans C Verstuyf A Mathieu Mathieu AC: Vitamin D's effect on immune function. Nutrients. (2020) 12:1248. 10.3390/nu12051248 32353972 PMC7281985

[51] Gnagnarella P Raimondi S Aristarco V Johansson HA Bellerba F Corso F. Vitamin D receptor polymorphisms and cancer. Adv Exp Med Biol. (2020) 1268:53–114. 10.1007/978-3-030-46227-7_4 32918214

[52] Canning MO Grotenhuis K de Wit H Ruwhof C Drexhage HA. 1-alpha,25-Dihydroxyvitamin D3 (1,25(OH)(2)D(3)) hampers the maturation of fully active immature dendritic cells from monocytes. Eur J Endocrinol. (2001) 145:351–7. 10.1530/eje.0.1450351 11517017

[53] Chun RF Liu NQ Lee T Schall JI Denburg MR Rutstein RM. Vitamin D supplementation and antibacterial immune responses in adolescents and young adults with HIV/AIDS. J Steroid Biochem Mol Biol. (2015) 148:290–7. 10.1016/j.jsbmb.2014.07.013 25092518 PMC4312738

[54] Ho LJ Wu CH Luo SF Lai JH. Vitamin D and systemic lupus erythematosus: causality and association with disease activity and therapeutics. Biochem Pharmacol. (2024) 227:116417. 10.1016/j.bcp.2024.116417 38996931

[55] Chen H Zhou Y Tang Y Lan J Lin C Chen Q. Neutrophil extracellular traps in tumor progression of gynecologic cancers. Front Immunol. (2024) 15:1421889. 10.3389/fimmu.2024.1421889 39555072 PMC11563837

[56] Ning Y Chen Y Tian T Gao X Liu X Wang J. S100A7 orchestrates neutrophil chemotaxis and drives neutrophil extracellular traps (NETs) formation to facilitate lymph node metastasis in cervical cancer patients. Cancer Lett. (2024) 605:217288. 10.1016/j.canlet.2024.217288 39384116

[57] Agraz-Cibrian JM Giraldo DM Urcuqui-Inchima S. 1,25-Dihydroxyvitamin D(3) induces formation of neutrophil extracellular trap-like structures and modulates the transcription of genes whose products are neutrophil extracellular trap-associated proteins: a pilot study. Steroids. (2019) 141:14–22. 10.1016/j.steroids.2018.11.001 30414422

[58] Molinari C Uberti F Grossini E Vacca G Carda S Invernizzi M. 1α,25-dihydroxycholecalciferol induces nitric oxide production in cultured endothelial cells. Cell Physiol Biochem. (2011) 27:661–8. 10.1159/000330075 21691084

[59] Shahidzadeh Yazdi Z Streeten EA Whitlatch HB Montasser ME Beitelshees AL Taylor SI. Critical role for 24-hydroxylation in homeostatic regulation of vitamin D metabolism. J Clin Endocrinol Metab. (2025) 110:e443–55. 10.1210/clinem/dgae156 38481375 PMC11747702

[60] Guo Y Li Y Tang Z Geng C Xie X Song S. Compromised NHE8 expression is responsible for vitamin D-deficiency induced intestinal barrier dysfunction. Nutrients. (2023) 15:4834. 10.3390/nu15224834 38004229 PMC10674576

[61] Liu FH Li SS Li XX Wang S Li MG Guan L Luan TG. Vitamin D3 induces vitamin D receptor and HDAC11 binding to relieve the promoter of the tight junction proteins. Oncotarget. (2017) 8:58781–9. 10.18632/oncotarget.17692 28938596 PMC5601692

[62] Chauss D Freiwald T McGregor R Yan B Wang L Nova-Lamperti E. Autocrine vitamin D signaling switches off pro-inflammatory programs of T(H)1 cells. Nat Immunol. (2022) 23:62–74. 10.1038/s41590-021-01080-3 34764490 PMC7612139

[63] Alvarez N Gonzalez SM Hernandez JC Rugeles MT Aguilar-Jimenez W. Calcitriol decreases HIV-1 transfer *in vitro* from monocyte-derived dendritic cells to CD4 + T cells, and downregulates the expression of DC-SIGN and SIGLEC-1. PLoS ONE. (2022) 17:e0269932. 10.1371/journal.pone.0269932 35802715 PMC9269915

[64] Villegas-Ospina S Aguilar-Jimenez W Gonzalez SM Rugeles MT. Vitamin D modulates the expression of HLA-DR and CD38 after *in vitro* activation of T-cells. Horm Mol Biol Clin Investig. (2017) 29:93–103. 10.1515/hmbci-2016-0037 28222027

[65] Bakdash G Schneider LP van Capel TM Kapsenberg ML Teunissen MB de Jong EC. Intradermal application of vitamin D3 increases migration of CD14+ dermal dendritic cells and promotes the development of Foxp3+ regulatory T cells. Hum Vaccin Immunother. (2013) 9:250–8. 10.4161/hv.22918 23291929 PMC3859743

[66] Zhang Q He X Chen W Jiu J Gao C Gao T. Vitamin D3 attenuates autoimmune thyroiditis by regulating Th17/Treg cell differentiation via YAP/JAK1/STAT1 axis. Immunol Lett. (2024) 269:106890. 10.1016/j.imlet.2024.106890 38959983

[67] Peelen E Thewissen M Knippenberg S Smolders J Muris AH Menheere P. Fraction of IL-10+ and IL-17+ CD8 T cells is increased in MS patients in remission and during a relapse, but is not influenced by immune modulators. J Neuroimmunol. (2013) 258:77–84. 10.1016/j.jneuroim.2013.02.014 23517930

[68] Thien R Baier K Pietschmann P Peterlik M Willheim M. Interactions of 1 alpha,25-dihydroxyvitamin D3 with IL-12 and IL-4 on cytokine expression of human T lymphocytes. J Allergy Clin Immunol. (2005) 116:683–9. 10.1016/j.jaci.2005.05.013 16159643

[69] Wang W Fu L Li S Xu Z Qiu P Xu TJ. Vitamin D insufficiency correlates with peripheral B10 cells in patients with pituitary tumours. Cell Biochem Funct. (2017) 35:254–9. 10.1002/cbf.3270 28749078

[70] Ai C. A study examining the correlation between serum 25-OH-VitD levels, CD3-CD19+ B lymphocytes, and the risk of early spontaneous abortion in pregnant women. Medicine. (2023) 102:e34338. 10.1097/MD.0000000000034338 37443483 PMC10344541

[71] Emmanouilidou G Kalopitas G Bakaloudi DR Karanika E Theocharidou E Germanidis G. Vitamin D as a chemopreventive agent in colorectal neoplasms. A systematic review and meta-analysis of randomized controlled trials. Pharmacol. Therap. (2022) 237:108252. 10.1016/j.pharmthera.2022.108252 35926664

[72] Vahedpoor Z Jamilian M Bahmani F Aghadavod E Karamali M Kashanian M. Effects of long-term vitamin D supplementation on regression and metabolic status of cervical intraepithelial neoplasia: a randomized, double-blind, placebo-controlled trial. Horm Cancer. (2017) 8:58–67. 10.1007/s12672-016-0278-x 28050798 PMC10355858

[73] Vahedpoor Z Mahmoodi S Samimi M Gilasi HR Bahmani F Soltani A. Long-term vitamin D supplementation and the effects on recurrence and metabolic status of cervical intraepithelial neoplasia grade 2 or 3: a randomized, double-blind, placebo-controlled trial. Ann Nutr Metab. (2018) 72:151–60. 10.1159/000487270 29466786

[74] Li D Liu Y Kong D Papukashvili D Rcheulishvili N Zhao H. Vitamin D receptor gene polymorphisms and the risk of CIN2+ in Shanxi population. Biomed Res Int. (2022) 2022:6875996. 10.1155/2022/6875996 36440356 PMC9683960

[75] Deuster E Jeschke U Ye Y Mahner S Czogalla B: Vitamin D and VDR in gynecological cancers—a systematic review. Int J Mol Sci. (2017) 18:2328. 10.3390/ijms18112328 29113037 PMC5713297

[76] Troja C Hoofnagle AN Szpiro A Stern JE Lin J Winer RL. Understanding the role of emerging vitamin D biomarkers on short-term persistence of high-risk human papillomavirus infection among mid-adult women. J Infect Dis. (2021) 224:123–32. 10.1093/infdis/jiaa711 33205195 PMC8491839

[77] Punchoo R Dreyer G Pillay TS: 25-hydroxycholecalciferol inhibits cell growth and induces apoptosis in SiHa cervical cells via autocrine vitamin D metabolism. Biomedicines. (2023) 11:871. 10.3390/biomedicines11030871 36979850 PMC10045786

[78] Xu C Liu J. Influence of vitamin D supplementation and the vaginal microenvironment on human papillomavirus infection. Afr J Reprod Health. (2024) 28:88–98. 10.29063/ajrh2024/v28i10.9 39625152

[79] Hernández-Rangel AE Hernandez-Fuentes GA Montes-Galindo DA Sanchez-Ramirez CA Cabrera-Licona A Martinez-Fierro ML. Vitamin D3 (calcitriol) monotherapy decreases tumor growth, increases survival, and correlates with low neutrophil-to-lymphocyte ratio in a murine HPV-16-related cancer model. Biomedicines. (2024) 12:1357. 10.3390/biomedicines12061357 38927564 PMC11201479

[80] Özgü E Yilmaz N Başer E Güngör T Erkaya S Yakut H. Could 25-OH vitamin D deficiency be a reason for HPV infection persistence in cervical premalignant lesions? J Exp Ther Oncol. (2016) 11:177–80. 28471122

[81] Wu Q Zhang L Sun Y Ying J. Vitamin D-regulated miR-589-3p in patients with cervical cancer predicts patient prognosis and is involved in tumor progression. Nutr Cancer. (2024) 76:840–8. 10.1080/01635581.2024.2365473 38913397

[82] Zhou E Bhoora S Pillay TS Punchoo R. Induction of cell death and regulation of autocrine vitamin D metabolism in cervical cancer by physiological and GI20 doses of 25-hydroxycholecalciferol. Int J Mol Sci. (2025) 26:4008. 10.3390/ijms26094008 40362248 PMC12071354

[83] Wang G Lei L Zhao X Zhang J Zhou M Nan K. Calcitriol inhibits cervical cancer cell proliferation through downregulation of HCCR1 expression. Oncol Res. (2014) 22:301–9. 10.3727/096504015X14424348425991 26629942 PMC7842578

[84] Friedrich M Meyberg R Axt-Fliedner R Villena-Heinsen C Tilgen W Schmidt W. Vitamin D receptor (VDR) expression is not a prognostic factor in cervical cancer. Anticancer Res. (2002) 22:299–304. 12017307

[85] Zhang Z Yu X Cheng G. Vitamin D sensitizes cervical cancer to radiation-induced apoptosis by inhibiting autophagy through degradation of Ambra1. Cell Death Discov. (2025) 11:1. 10.1038/s41420-024-02279-7 39753527 PMC11698873

[86] Bhoora S Pather Y Marais S Punchoo R: Cholecalciferol Inhibits Cell Growth and Induces Apoptosis in the CaSki Cell Line. Med Sci. (2020) 8:12. 10.3390/medsci8010012 32069830 PMC7151577

[87] Faryabi A Salari MA Dalvand A Akbarniakhaky H Mohammadi G Aazami H. Mapping the landscape of vitamin D in cancer studies: a systematic global investigation. J Diabetes Metab Disord. (2025) 24:78. 10.1007/s40200-025-01594-9 40078705 PMC11893971

[88] Salih MM Almehmadi M Shafie A Alsharif A Alsiwiehri N El-Askary A. Evaluation of CD4+:CD8+ ratio in patients with cervical cancer and the levels of inflammatory markers. In vivo. (2022) 36:2414–21. 10.21873/invivo.12975 36099148 PMC9463885

[89] Vijayendra Chary A Hemalatha R Seshacharyulu M Vasudeva Murali M Jayaprakash D Dinesh Kumar B. Vitamin D deficiency in pregnant women impairs regulatory T cell function. J Steroid Biochem Mol Biol. (2015) 147:48–55. 10.1016/j.jsbmb.2014.11.020 25448751

[90] Ma JG Wu GJ Xiao HL Xiao YM Zha L. Vitamin D has an effect on airway inflammation and Th17/Treg balance in asthmatic mice. Kaohsiung J Med Sci. (2021) 37:1113–21. 10.1002/kjm2.12441 34460994 PMC11896363

[91] Kloc M Ghobrial RM Lipińska-Opałka A Wawrzyniak A Zdanowski R Kalicki B. Effects of vitamin D on macrophages and myeloid-derived suppressor cells (MDSCs) hyperinflammatory response in the lungs of COVID-19 patients. Cell Immunol. (2021) 360:104259. 10.1016/j.cellimm.2020.104259 33359760 PMC7738277

[92] Stachowicz-Suhs M Łabedz N Milczarek M Kłopotowska D Filip-Psurska B Maciejczyk A. Vitamin D(3) reduces the expression of M1 and M2 macrophage markers in breast cancer patients. Sci Rep. (2024) 14:22126. 10.1038/s41598-024-73152-x 39333342 PMC11437092

[93] Dionne S Duchatelier CF Seidman EG. The influence of vitamin D on M1 and M2 macrophages in patients with Crohn's disease. Innate Immun. (2017) 23:557–65. 10.1177/1753425917721965 28770666

[94] Cheung MM Dall RD Shewokis PA Altasan A Volpe SL Amori R. The effect of combined magnesium and vitamin D supplementation on vitamin D status, systemic inflammation, and blood pressure: a randomized double-blinded controlled trial. Nutrition. (2022) 99-100:111674. 10.1016/j.nut.2022.111674 35576873

[95] Gonçalves de. Carvalho CM, Ribeiro SM: Aging, low-grade systemic inflammation and vitamin D: a mini-review. Eur J Clin Nutr. (2017) 71:434–40. 10.1038/ejcn.2016.177 27677370

[96] Giuggioli D Colaci M Cassone G Fallahi P Lumetti F Spinella A. Serum 25-OH vitamin D levels in systemic sclerosis: analysis of 140 patients and review of the literature. Clin Rheumatol. (2017) 36:583–90. 10.1007/s10067-016-3535-z 28070764

[97] Wang W Hu W Xue S Chen Q Jiang Y Zhang H. Vitamin D and lung cancer; association, prevention, and treatment. Nutr Cancer. (2021) 73:2188–200. 10.1080/01635581.2020.1844245 33225744

[98] Bellerba F Serrano D Harriet J Pozzi C Segata N NabiNejad A. Colorectal cancer, vitamin D and microbiota: a double-blind Phase II randomized trial (ColoViD) in colorectal cancer patients. Neoplasia. (2022) 34:100842. 10.1016/j.neo.2022.100842 36279751 PMC9594107

[99] Gwenzi T Schrotz-King P Schöttker B Hoffmeister M Brenner H. Vitamin D status, *Cdx2* genotype, and colorectal cancer survival: population-based patient cohort. Nutrients. (2023) 15:2717. 10.3390/nu15122717 37375621 PMC10305330

[100] Arayici ME Basbinar Y Ellidokuz H. Vitamin D intake, serum 25-hydroxyvitamin-D (25(OH)D) levels, and cancer risk: a comprehensive meta-meta-analysis including meta-analyses of randomized controlled trials and observational epidemiological studies. Nutrients. (2023) 15:2722. 10.3390/nu15122722 37375626 PMC10302100

[101] Renke G Starling-Soares B Baesso T Petronio R Aguiar D Paes R. Effects of vitamin D on cardiovascular risk and oxidative stress. Nutrients. (2023) 15:769. 10.3390/nu15030769 36771474 PMC9920542

[102] Kimball SM Holick MF. Official recommendations for vitamin D through the life stages in developed countries. Eur J Clin Nutr. (2020) 74:1514–8. 10.1038/s41430-020-00706-3 32820241

[103] Rusu ME Bigman G Ryan AS Popa DS. Investigating the effects and mechanisms of combined vitamin D and K supplementation in postmenopausal women: an up-to-date comprehensive review of clinical studies. Nutrients. (2024) 16:2356. 10.3390/nu16142356 39064799 PMC11279569

[104] Hu Z Zhang H Yi B Yang S Liu J Hu J. VDR activation attenuate cisplatin induced AKI by inhibiting ferroptosis. Cell Death Dis. (2020) 11:73. 10.1038/s41419-020-2256-z 31996668 PMC6989512

[105] Lu D Yu M Chen L Ye J Huang L Zhu G. EB1089 promotes the expression of vitamin D receptor in the intestinal epithelial cell line HT-29 and reduces lipopolysaccharide-induced inflammatory response. Ann Transl Med. (2022) 10:476. 10.21037/atm-22-1066 35571391 PMC9096417

[106] Wang H Yu X Liu D Qiao Y Huo J Pan S. VDR activation attenuates renal tubular epithelial cell ferroptosis by regulating Nrf2/HO-1 signaling pathway in diabetic nephropathy. Adv Sci. (2024) 11:e2305563. 10.1002/advs.202305563 38145959 PMC10933633

[107] Ishikawa LLW Colavite PM Fraga-Silva TFC Mimura LAN França TGD Zorzella-Pezavento SFG. Vitamin D deficiency and rheumatoid arthritis. Clin Rev Allergy Immunol. (2017) 52:373–88. 10.1007/s12016-016-8577-0 27484684

[108] Zhang Y Wang C Zhang L Yu J Yuan W Li L. Vitamin D(3) eradicates Helicobacter pylori by inducing VDR-CAMP signaling. Front Microbiol. (2022) 13:1033201. 10.3389/fmicb.2022.1033201 36569092 PMC9772467

[109] Tsuji T Hiroyuki A Uraki S Doi A Morita S Iwakura H. Autosomal dominant hypocalcemia with atypical urine findings accompanied by novel *CaSR* gene mutation and VitD deficiency. J Endocrine Soc. (2021) 5:bvaa190. 10.1210/jendso/bvaa190 33506158 PMC7814383

[110] Oussaada SM Akkermans I Chohan S Limpens J Twisk JWR Winkler C. The effect of active vitamin D supplementation on body weight and composition: a meta-analysis of individual participant data. Clin Nutr. (2024) 43:99–105. 10.1016/j.clnu.2024.08.031 39357088

[111] Lang PO Aspinall R. Can we translate vitamin D immunomodulating effect on innate and adaptive immunity to vaccine response? Nutrients. (2015) 7:2044–60. 10.3390/nu7032044 25803545 PMC4377899

[112] Luo J Chen H Ma F Xiao C Sun B Liu Y. Vitamin D metabolism pathway polymorphisms are associated with efficacy and safety in patients under anti-PD-1 inhibitor therapy. Front Immunol. (2022) 13:937476. 10.3389/fimmu.2022.937476 36172344 PMC9510606

[113] Khamis A Gül D Wandrey M Lu Q Knauer SK Reinhardt C. The vitamin D receptor-BIM axis overcomes cisplatin resistance in head and neck cancer. Cancers. (2022) 14:5131. 10.3390/cancers14205131 36291915 PMC9600548

[114] Clark AS Chen J Kapoor S Friedman C Mies C Esserman L. Pretreatment vitamin D level and response to neoadjuvant chemotherapy in women with breast cancer on the I-SPY trial (CALGB 150007/150015/ACRIN6657). Cancer Med. (2014) 3:693–701. 10.1002/cam4.235 24719175 PMC4101761

[115] Gong T Sun R Bai J Liu X He C Jiang Q. Calcitriol modulates hippocampal axon guidance through enhanced EfnA4-Mediated PI3K/AKT signaling in an autism mouse model. CNS Neurosci Ther. (2025) 31:e70429. 10.1111/cns.70429 40395150 PMC12093051

[116] Battistini C Ballan R Herkenhoff ME Saad SMI Sun J. Vitamin D modulates intestinal microbiota in inflammatory bowel diseases. Int J Mol Sci. (2020) 22:362. 10.3390/ijms22010362 33396382 PMC7795229

[117] Fraga M Yáñez M Sherman M Llerena F Hernandez M Nourdin G. Immunomodulation of T helper cells by tumor microenvironment in oral cancer is associated with CCR8 expression and rapid membrane vitamin D signaling pathway. Front Immunol. (2021) 12:643298. 10.3389/fimmu.2021.643298 34025655 PMC8137990

